# Nanoparticle formulations for therapeutic delivery, pathogen imaging and theranostic applications in bacterial infections

**DOI:** 10.7150/thno.82790

**Published:** 2023-03-05

**Authors:** Lai Jiang, Linyu Ding, Gang Liu

**Affiliations:** State Key Laboratory of Molecular Vaccinology and Molecular Diagnostics & Center for Molecular Imaging and Translational Medicine, School of Public Health, Xiamen University, 361102, China.

**Keywords:** Nanoparticles, Therapeutic delivery, Diagnosis, Theranostic, Bacterial Infection

## Abstract

Pathogenic bacterial infections represent an ever-growing crisis, now significantly threatening life expectancy across the worldwide population and thus novel approaches to tackle this issue are urgently needed. The application of nanotechnology in recent years has opened up new horizons in the selective or specific delivery of drugs or imaging agents to infectious sites. In particular, the development of nanoparticles for both delivery of active substances and imaging of infection sites is now gathering much interest. Although still in its infancy, the field of antibacterial nanomedicines provides exciting new possibilities to combat multi-resistant bacterial infections and shows great promise for personalized medicine in antibacterial stewardship. This review examines nanoparticle-based formulations used for therapeutic delivery, pathogen tracking in diagnosis, and combined “theranostic” approaches to more effectively treating bacterial infections.

## Introduction

Infectious diseases caused by a causative agent, a bacterium, parasite, or virus, represent a growing international health risk, as over 50% of global deaths each year can be ascribed to infectious agents 1, 2. However, the incidence of microorganisms with drug resistance has grown due to the widespread usage of antibiotics over the past 70 years, which is now a worldwide severe health concern 3, 4. Inefficiencies further exacerbate this problem with antibiotics, including low bioavailability, short half-life, side effects, and poor permeability of cellular barriers and biofilms 5, 6. Therefore, researchers have increasingly evaluated drug delivery systems and other antimicrobial materials to improve the shortcomings associated with current antibiotics to enhance their effectiveness and prevent the development of resistance in patients 7-11.

The bio-application of nanotechnology has been extensively examined to develop more effective therapeutic delivery platforms 12, 13, including formulations such as liposomes 14-17, dendrimers 18-20, nano-emulsions 21, 22, polymeric 23-26 and metallic nanoparticles 27, 28. These modalities, coupled with the ability to manipulate their features, excipients, and size, provide the ability to develop formulations that can be optimized to efficiently encapsulate drugs of different solubilities, tailor/control their release, and generate more targeted effects. The apparent superiority of nanoparticle delivery technology in antimicrobial therapy is its ability to tackle existing drug resistance, avoiding further development and adverse effects. Moreover, by inhibiting the biofilm formation and targeting intracellular microbes, nanoparticle delivery system also presents their unique advantages. More importantly, some nanoparticles also “deliver” therapeutic effects through their antimicrobial properties, such as antimicrobial polymeric nanoparticles 29, reactive oxygen species-producing metallic nanoparticles 30, and antimicrobial photothermal therapy-inducing carbon nanotubes 31, 32.

In addition to their obvious application as therapeutic delivery systems, nanoparticles are promising platforms for developing imaging modalities. Magnetic nanoparticles 33-35, gold nanoparticles 36-38, and fluorescent nanoparticles have all been employed for microbiological diagnostics. In contrast to small molecule probes, which are likely to be inactivated by non-specific binding of substances *in vivo*, nanoparticle-based probes or imaging modules can significantly extend the retention duration of imaging moieties *in vivo* and thus improve imaging quality with time. In addition, nanoparticles may be easily absorbed by cells, for example showing a very pronounced EPR (enhanced permeability and retention) effect, whereas small molecule probes virtually ever do. More critically, with the advancement of nanoparticle surface engineering, sensitive and specific detection for pathogenic bacteria could be easily achieved through binding nanoparticles with affinity probes, such as antibodies. In the recent ten years, particularly, there has been significant advancement in the development of nanotechnology-based diagnostics, resulting in the production of goods like Feraheme® 39.

**Figure 1:**
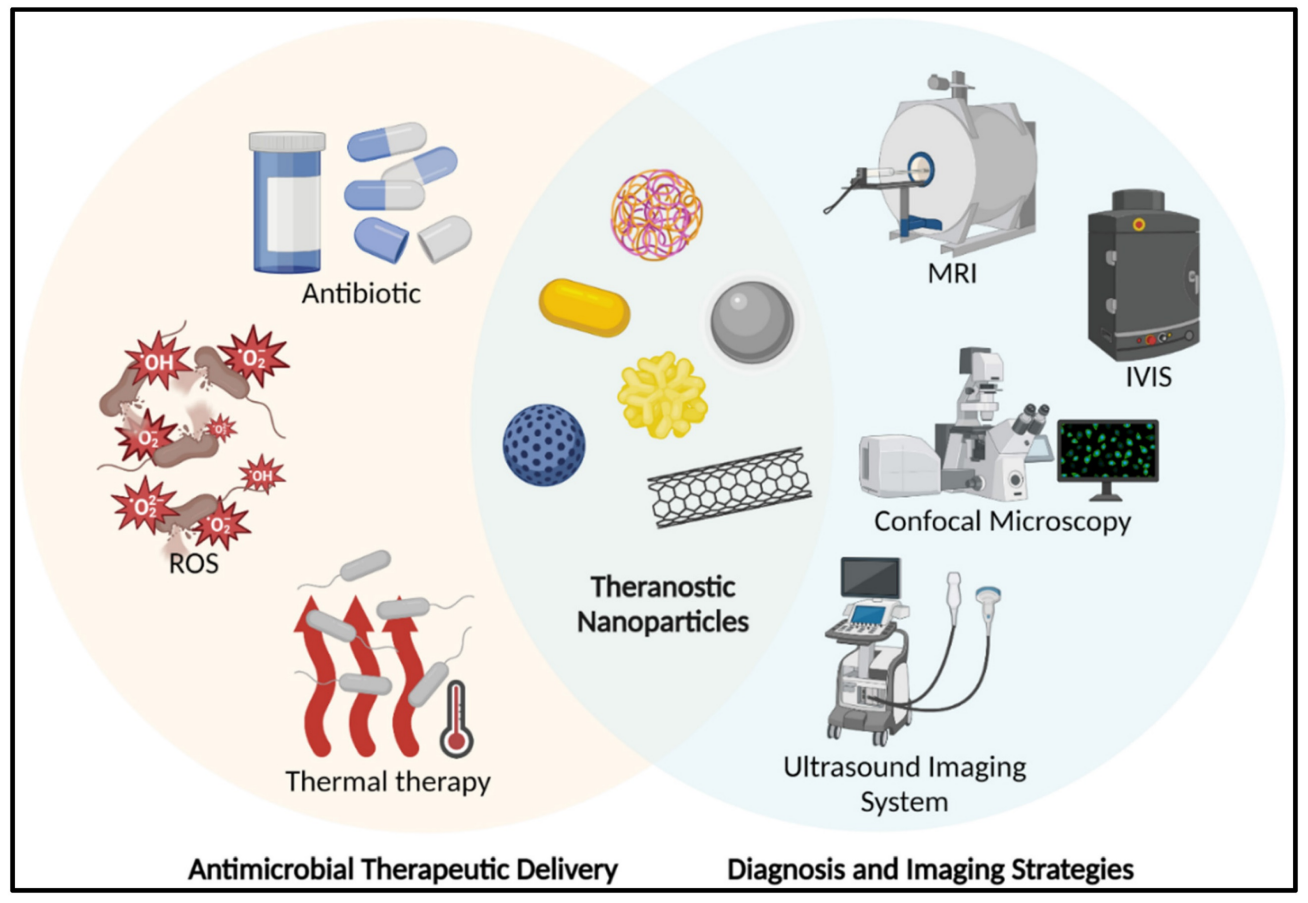
A schematic represents the theranostic nanoparticles in anti-bacterial applications. (MRI: Magnetic Resonance Imaging; IVIS: In Vivo Imaging System; ROS: Reactive Oxygen Species). Created with BioRender.com.

The ultimate aim of many researchers in the nanotechnology arena is to develop nanomaterials that have both therapeutic and diagnostic potential; the coined “theranostic” modality represents the holy grail 40-42. Conceptually, a theranostic combines diagnostic and therapeutic utility as a single formulation/modality **(Figure 1)**. There are several well-established, effective, and safe theranostic anti-cancer therapies, such as lutetium octreotate 43 for somatostatin-positive tumour cells and Iodine-131 therapy 44 for thyrotoxicosis and thyroid cancer. However, given the complexity required to develop such formulations, proposed theranostic nanoparticle anti-infection strategies are currently in incipient stages. The next sections will go through the several recent nano-formulations that have successfully completed approaches for the treating and diagnostic imaging of microbial infections as well as the potential for integrating theranostic nanoparticles.

## Lipid-based Nano-formulations

### Lipid-based nano-formulations as antimicrobial deliver systems

Liposomes represent the most developed and widely applicable drug delivery platform. They are composed of phospholipid bilayers that combine to create a spherical vesicle with an aqueous centre. Liposomes have an amphiphilic structure that allows them to transport hydrophobic substances into their bilayers or encapsulate hydrophilic molecules in their core. As the most developed drug delivery system, numerous liposome formulations have successfully completed clinical evaluation and many liposome formulations have also been commercialized successfully. For example, Arikayce®, composed of DPPC (1,2-dipalmitoyl-sn-glycero-3-phosphocholine) and cholesterol, is an inhaled liposomal amikacin formulation, which is the first and only therapy approved in the U.S. specifically for patients with MAC (mycobacterium avium complex) lung illness 45, 46.

Recent studies have investigated liposomal DDS (drug delivery system) for treating various bacterial infections, suggesting that liposomal formulations that would deliver antibiotics to the site of action with a controlled release could significantly improve therapeutic utility 47-49. An example of this is the addition of molecules such as oleic acid derived quaternary lipid, whose structural conformation changes under acidic conditions, causes the liposomes to disintegrate and release the contained drug 50. Such acid-responsive formulations are well adapted to the weakly acidic inflammatory microenvironment in which bacterial infections occurred. Then there are some thermosensitive liposomal molecules (e.g. DPPC, DSPC), which maintain a stable liposomal structure under physiological conditions and can trigger drug release in association with topical thermotherapy which can also inactivate bacteria through thermal effects, making them applicable to the treatment of wound infections in superficial tissues 51-53.

Liposome-based formulations provide an expansive range of opportunities to deliver therapies to generate synergistic effects. However, some drawbacks, such as short circulation half-life, low encapsulation efficacy of water-soluble drugs, and uncontrollable fusion, have plagued liposomal vesicles. To solve these pharmacological challenges, liposomes can be modified with a surface coat of PEG (polyethylene glycol) 54, gangliosides 55-57 and sialic acid derivatives 58, 59 to limit opsonization by the reticuloendothelial system and attenuate this rapid clearance from blood circulation. Drug encapsulation can be improved by introducing new steps, such as the dehydration-rehydration technique in the liposome formulation process 60. As for improving the stability against uncontrollable fusion among liposomes, some studies reported that cationic and/or anionic nanoparticles could be decorated onto liposomes to enhance stability effectively 61. Pornpattananangkul *et al.* synthesized a liposome stabilized by gold nanoparticles (modified with chitosan) 62. They demonstrated that the attaching of gold nanoparticles to liposomes may successfully block fusion between liposomes to prevent undesirable vancomycin release. Moreover, the triggered release was achieved by inserting toxins secreted by *S. aureus* (*Staphylococcus aureus*) into liposomal layers and forming pores to release the encapsulated vancomycin. This novel approach has been proven to be applied in treating infection caused by toxin-secreting bacteria.

**Figure 2:**
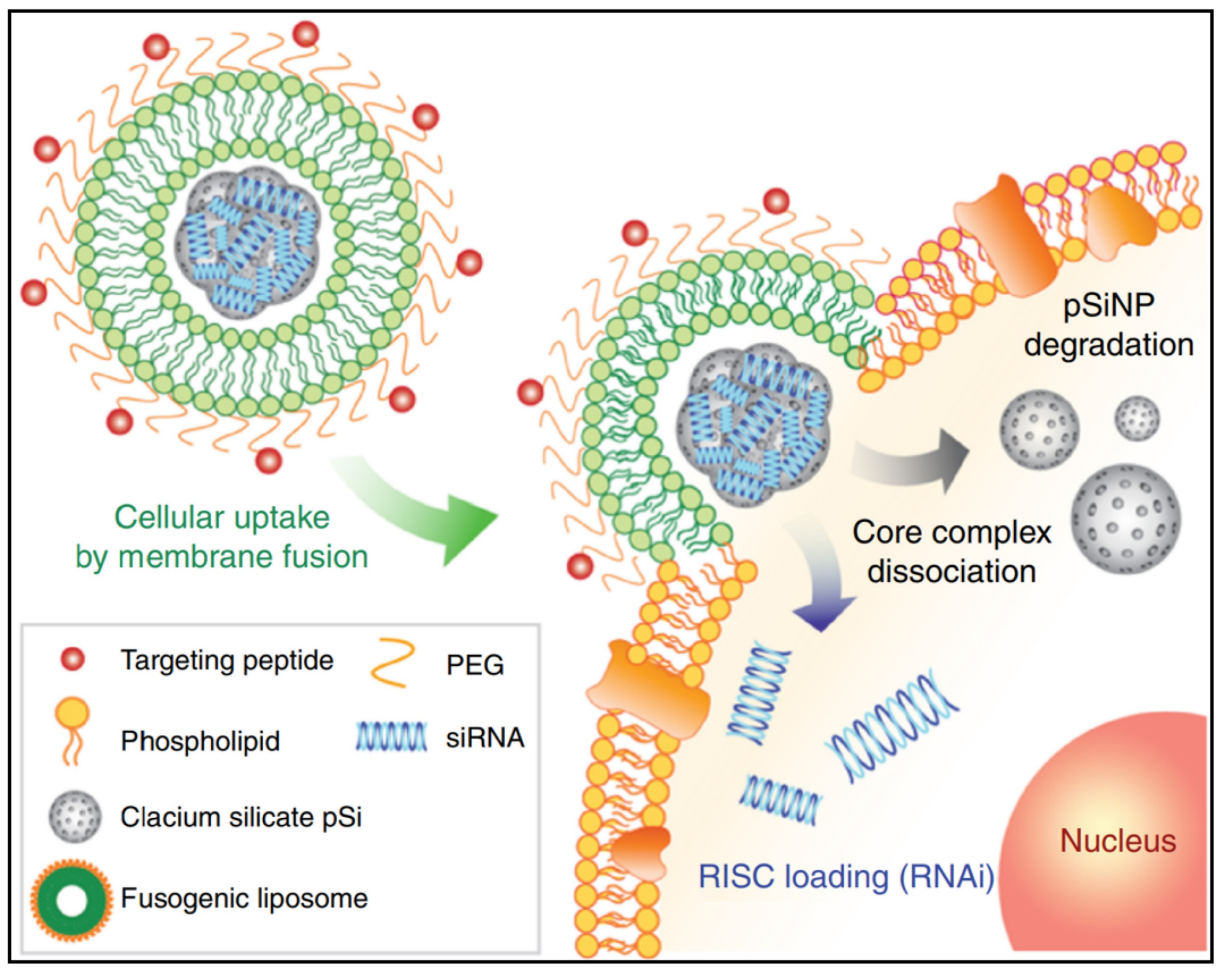
A schematic presents the mechanism of the fusogenic pSiNP. Copyright © 2018 Springer Nature

siRNA (small interfering RNA) is an alternative approach to fight bacterial infections by enhancing phagocytosis and bacterial clearance of macrophages. For instance, Niu *et al.* created liposomes loaded with siRNA against TGF-β1 (transforming growth factor-β1) that could target *M. tuberculosis* (*Mycobacterium tuberculosis*)-infected host THP-1 macrophages and enhance their autophagy 63. In such siRNA therapies, the effectiveness of RNA interference is crucial. However, in conventional methods, such as nanoparticles loaded with siRNA, most of the nanoparticles are taken up via endocytosis of cells and then remain in the low pH endosomal compartment, which is later fused by lysosomes to form late endosomes, where they are degraded. Furthermore, lysosomes contain nucleases that degrade siRNA, thus no RNA interference can occur if the loaded siRNA cannot be leaked and excreted into the cytoplasm during early endosome uptake 64-66. This is the most significant problem faced by RNA delivery systems for intracellular transport. To overcome this deficiency and improve gene therapy, Kim *et al*. developed a combinatorial system, nanoporous particles incorporated with fusogenic liposomes and peptides in the outer layer **(Figure 2)** 67. In this nano-system, the high colocalization of the DiI and FAM signals in the fusogenic formulation suggests that CRV binds the nanoparticles to the particular macrophage membrane receptors to facilitate localized membrane fusion. By transferring hydrophobic molecules from the lipid bilayer to the cellular membranes, this technique enables the hydrophilic payload (siRNA) to be released immediately from the silica NP through into cell cytoplasm. This efficient delivery system was shown to be successful in enhancing clearance and macrophage survival in an *S. aureus* infection model.

SLNs (solid lipid nanoparticles) have attracted increasing interest because these formulations have the opportunity to achieve excellent drug loading. For instance, in a formulation created by Vieira *et al.*, the encapsulation efficacy of rifampicin was approximately 90% 68. In addition, these anhydrous formulations may offer advantages in terms of long-term stability and biocompatibility. A particular property of SLN technology is the ability to prepare combination drug formulations more easily and uniformly than other drug delivery platforms. This is particularly important for infections such as* M. tuberculosis* that require combination therapy. Compared to conventional antibiotic administration, the creation of SLNs packed with isoniazid, pyrazinamide, and rifampicin via emulsion solvent diffusion approach showed a considerable improvement in antibiotic residence duration and dosage 69. No *M. tuberculosis* was detected in organs after daily nebulized administration of the multidrug SLNs over seven days; this is an astounding result, considering that regular oral administration of these drugs would require 46 daily doses to achieve equivalent therapeutic effects.

### Lipid-based nano-formulations as bacteria-labelling platforms

The accurate determination of bacterial load and infection foci is of significant importance. This is particularly relevant considering bacterial and sterile inflammation symptoms can appear very similar; thus, it is critically important to distinguish these to make appropriate clinical decisions. Targeting the leukocytes that migrate and localize at the infected site is widely considered a gold standard for imaging infections 70. However, there are limitations for labelled leukocytes in diagnostic accuracy, and the technique is time-consuming and can be hazardous to the patient. Therefore, more effective bacteria-imaging strategies are needed, which could directly label the invading pathogen instead of targeting surrogate micro-environmental factors. One promising approach is to attempt to tag the bacteria with fluorescent probes 71. Such a route may provide a convenient approach to visualizing infection in real-time. With these aspirations in mind, nanotechnology has brought a new perspective to labelling specific molecules or biomarkers. For example, liposomes have been exploited and utilized in bacteria-related bio-labelling 72-74. Fluorescence-based liposomes made from cholesterol, DPPC, DPPE, DPPG, and SRB were synthesized and functionalized with rabbit anti-*C. muytjensii (Cronobacter muytjensii)* IgG on its surface. This liposomal immunoassay provided directly proportional signal intensity to the number of *C. muytjensii*, suggesting this approach could offer a rapid and effective tool for *C. muytjensii* diagnosis 75.

**Figure 3:**
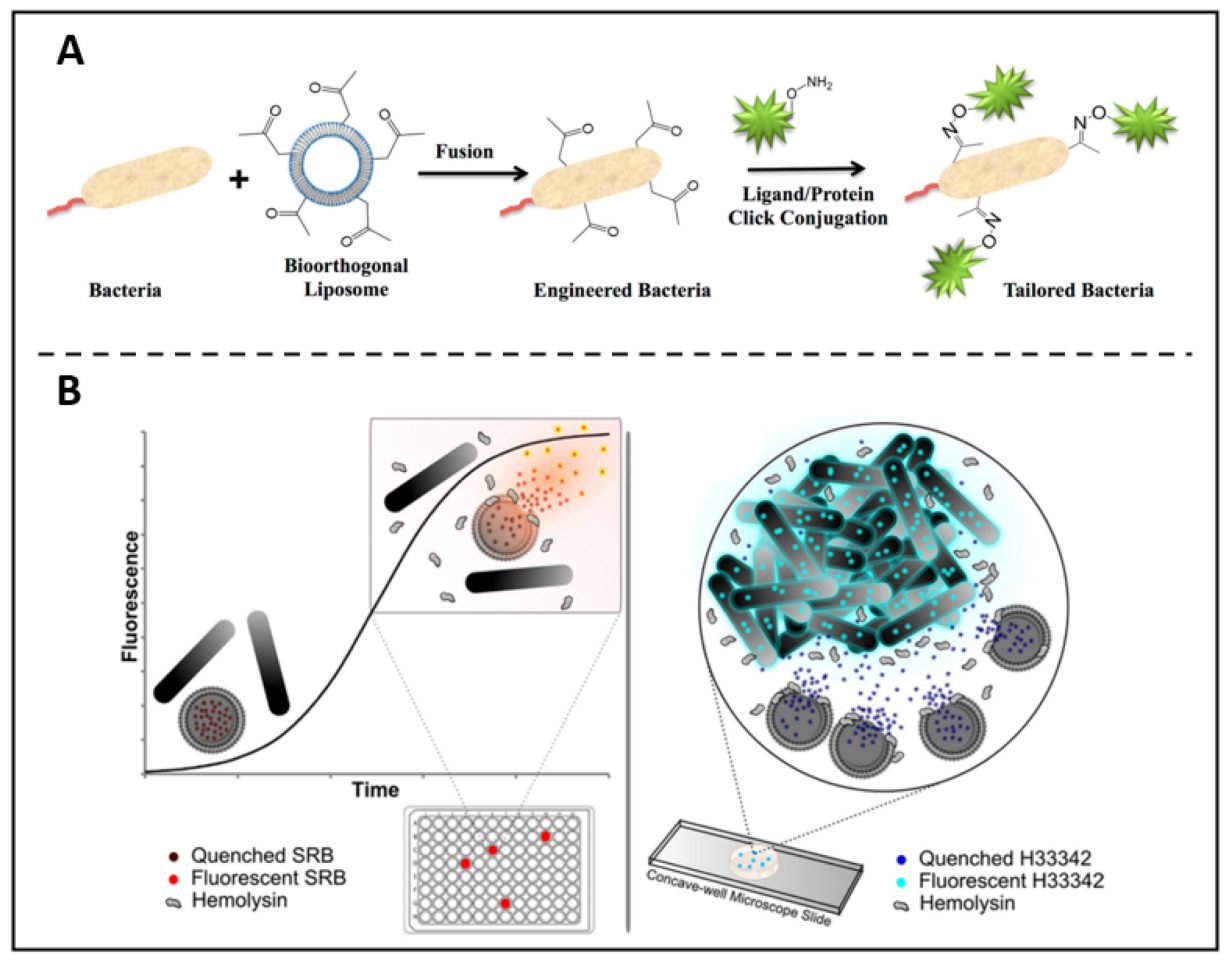
** (A)** Bio-orthogonal chemistry is used to quantitatively characterise the surface engineering of bacteria. Copyright © 2016 American Chemical Society **(B)** Liposome lysis is selectedly initiated by β-haemolytic bacteria, allowing quick and accurate pathogen identification. Copyright © 2017 American Chemical Society

In addition to being a fluorescent material that can directly label bacteria, liposomes have a number of properties that can be used as a precursor step to biolabeling. For example, based on the liposome-cell membrane fusion, Elahipanah *et al*. presented a bacteria-labelling strategy through the bio-orthogonal chemistry **(Figure 3A)** 76. In this work, by incubating with ketone-functionalized liposomes, in the presence of membrane fusion, ketone moieties could be presented on the bacterial membrane surface, which can subsequently be anchored by bio-orthogonal reactions with oxyamine tailored fluorescent molecules, proteins and probes that act as bio-markers. In another work, Sum *et al.* found that certain specific components of liposomes could be decomposed by β-hemolysin 77. They then loaded fluorophores within the liposomes and co-incubated with bacteria. In contrast to α- and γ-haemolytic bacteria, it was shown that β-haemolytic pathogens could lyse and release fluorophores that were enclosed in liposomes. This enables the detection and separation of certain bacteria with extreme sensitivity and accuracy **(Figure 3B)**.

### LIPID-based nano-formulations as antibacterial theranostics

**Figure 4:**
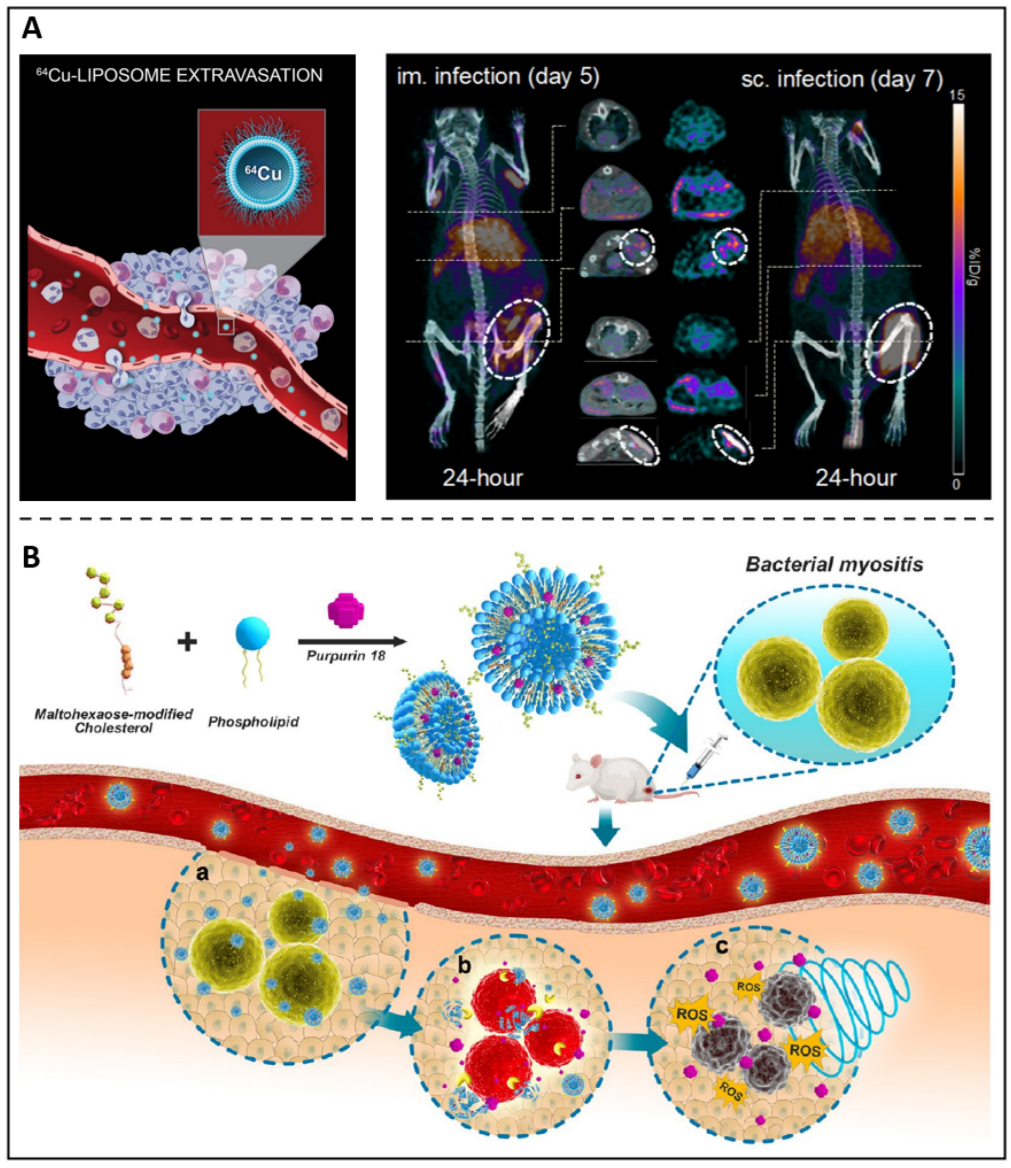
** (A)** A schematic presents ^64^Cu-liposome; PET/CT images of *S. aureus* Xen29 infection and injected with ^64^Cu-liposomes. Copyright © 2020 Elsevier B.V. **(B)** A schematic depicts MLP18 nanoliposomes for MRSA identification and elimination. Copyright © 2019 American Chemical Society.

Because of their chemical versatility in labelling for imaging needs and their capacity to transport antimicrobial medicines, liposomes constitute an appealing theranostic approach for treating microbial infections. Kaul *et al.* synthesized a folate receptor targeting, pegylated liposomal formulation with rifampicin and ofloxacin encapsulated to improve the therapeutic window towards mycobacterial infections 78. Additionally, the antibiotic-loaded liposomes were conjugated with a gamma-emitting radionuclide, thus providing diagnostic capabilities and presenting an effective and promising theranostic agent against tuberculosis. Clausen *et al.* developed an intramuscular and subcutaneous *S. aureus-induced* infection model using a bioluminescent strain (*S. aureus* Xen29) to examine the possibility of Cu-64 liposomes (Figure 4A) as a therapeutic approach to diagnose and evaluate patients who could achieve improved antibiotic therapy 79. Images revealed a distinct luminous signal in the injection location that diminished throughout the period, suggesting that the mice were capable of mounting an immune response. On 24-hour PET images, ^64^Cu-liposomes showed high activity at both intramuscular and subcutaneous infection sites of the inoculum. Due to the extremely high liposome activity, infectious lesions may benefit from using liposomes as a diagnostic imaging and pharmaceutical delivery system. We previously reported a sonotheranostic strategy, based on purpurin 18-loaded and maltohexaose-decorated nanoliposome (MLP18), for bacteria-specific labelling and the visualization of sonodynamic therapy (Figure 4B) 80. After entering the body, the prepared nano-liposomes can accurately stay at the site of bacterial infection and produce fluorescence / photoacoustic labelling to distinguish the lesions from joint inflammation or tumours. This vigorous acoustic dynamic activity was observed by *in situ* MRI imaging, which also showed that MLP18-mediated sonodynamic therapy effectively eliminated inflammation and abscesses in animals of microbial myositis.

Liposomes are extensively regarded and investigated as lipid-based drug delivery vehicles, not only due to the fundamental raw material for their manufacture, phospholipids, is an inherent element in human cells, making them friendly and non-immunogenic, but also since they can be manufactured as nanoscale particles, making them simpler to cross intrinsic limitations like blood vessel barriers and cellular membranes. In addition to antibacterial medication delivery, they are now employed in a variety of purposes, including as the treatment of cancer 81-83, eye illnesses 84, 85, and neurodegenerative diseases 86, 87.

## Polymeric Nano-formulations

### Antibiotic-loaded polymeric nanoparticles

The most common approach to preparing polymeric systems containing antibiotics is to load the antibiotics in the form of a physical encapsulation in polymeric particles. polymeric nanoparticles have the potential to be endocytosed intact by cells and bacteria, and successfully deliver the contained therapeutic agent into the cell, avoiding confinement at the cell membrane. This strategy improves both the solubility of hydrophobic antibiotics and the intracellular uptake of hydrophilic antibiotics, enhancing the antimicrobial efficacy to eliminate bacterial infections. In-depth research has been conducted on the exploitation of various biocompatible polymers, either natural or manufactured, as the building blocks for this nanosystem.

Due to their biocompatibility and biodegradability, natural polymers, including chitosan, alginate, and albumin, have been investigated as drug carriers 88-97. For example, ceftriaxone-loaded chitosan tripolyphosphate nanoparticles exhibited enhanced antibacterial effects against intracellular *S. typhimurium (Salmonella typhimurium)* than the inefficient free drug 98. Tobramycin-loaded alginate/chitosan nanoparticles engineered with DNase alfa were produced by Deacon and colleagues, which exhibited improved therapeutic effects against *P. aeruginosa (Pseudomonas aeruginosa)* in cystic fibrosis sputum, through at least in part, degradation of the extracellular DNA characteristically found in the sputum of these patients 99. In addition to these natural polymer-based nanoparticles, many artificial polymers have been used as delivery system material. The most investigated polymer for the targeted administration of antimicrobials into infected cells is PLGA (poly(lactic-co-glycolic acid)) 100-107. Radovic-Moreno *et al.* described the possibilities for a triblock copolymer of poly(d,l-lactic-co-glycolic acid)-b-poly(L-histidine)-b-poly(ethylene glycol) (PLGA-PLH-PEG) based on PLGA to self-assemble into nanostructures and encapsulate vancomycin in aqueous medium 108. Other synthetic polymers used in products approved by the FDA have also been investigated as drug delivery platforms to treat bacterial infections. For example, PCL (polycaprolactone) is highly suitable for colloidal drug delivery as it can entrap various drug agents readily and has low toxicity properties 104, 109, 110. A novel PCL-pluronic nanoparticle delivery platform was developed to improve the antibacterial effects of chloramphenicol, which further showed noticeably improved anti-MRSA (methicillin-resistant *Staphylococcus aureus*) effectiveness against a range of clinical specimens while dramatically reducing its toxicity 111. Copolymer based on PCL, for example, poly(ethyleneglycol)-b-polycaprolactone-b-poly(β-amino ester) (PEG-b-PCL-b-PAE) has been reported by Chu and co-workers 112. The bacterial infection-induced local acidity generated a charge transition in this self-assembled polymeric nano-micelle, prompting the encapsulated vancomycin to release and eradicate the bacteria at the site of the subcutaneous infection.

### Antibiotic-conjugated polymeric nanoparticles

**Figure 5:**
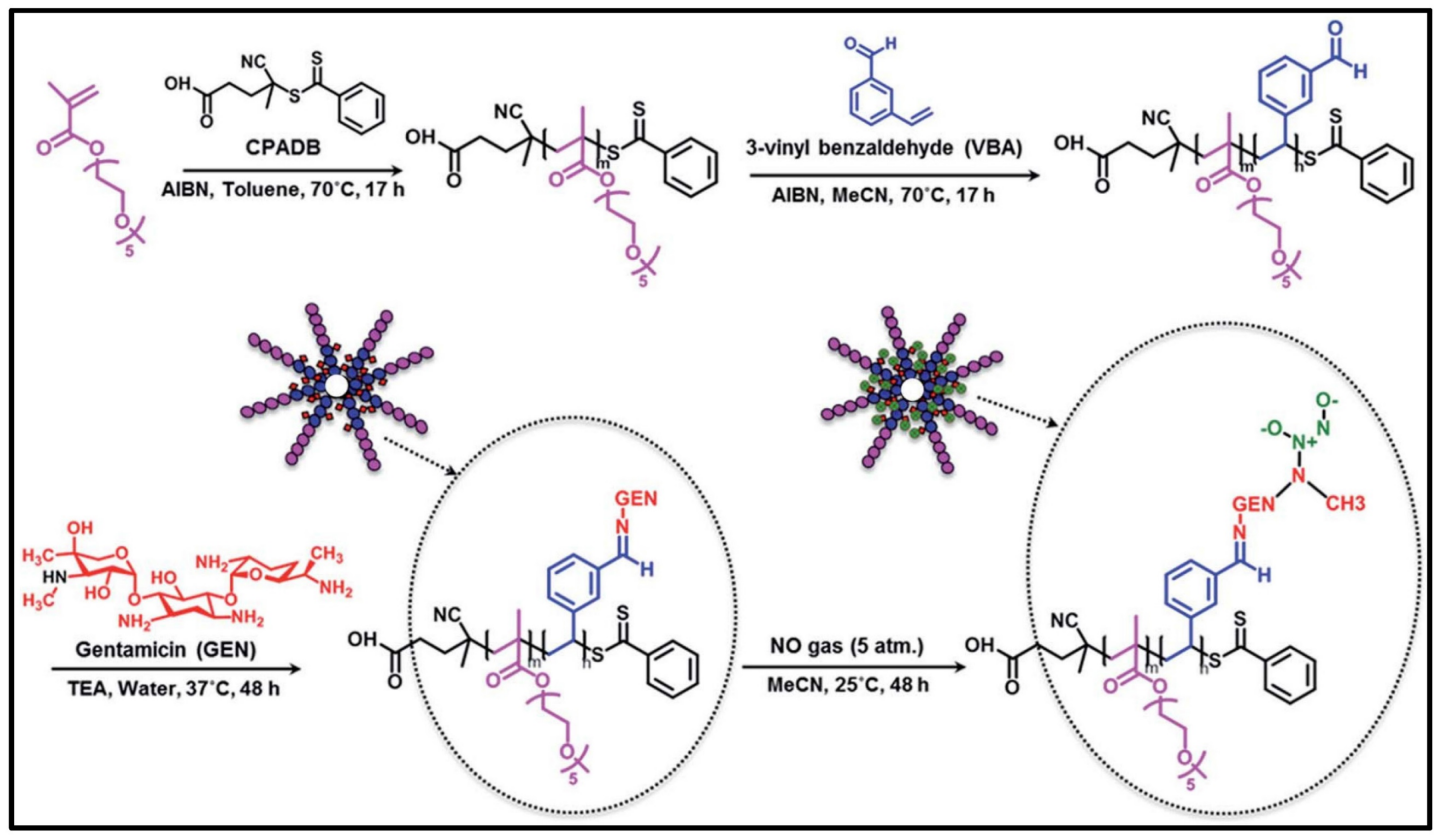
Schematic approach showing the RAFT polymerization is used in the manufacture of gentamicin-NONOate nanoparticles. Copyright © 2016 The Royal Society of Chemistry

Although encapsulation of antibiotic drugs in polymeric nanomicelles and nanoparticles has been successful in reducing bacterial loads and mitigating bacterial infections, it is undesirable for loaded medications to release prematurely or in an unintended manner, particularly hydrophilic antibiotics such as β-lactams, aminoglycosides, and sulphonamides. To get around this restriction, antibiotic molecules can be chemically bonded to polymers using a variety of chemical linkages, including amides, esters, ethers and carbamates, to form antibiotic-polymer conjugates, thus forming nanoparticles. Dendrimers, as one representative of this drug-polymer conjugation, are repetitively branched polymeric molecules with spherical three-dimensional morphology. Unlike polymeric nano-systems, a particular advantage of dendrimers is that drug molecules can be engineered as an integral part of the carrier through covalent conjugation, so that the payload could be delivered in a controlled way by the linkage or through some triggering events at the desired location 113-116. For instance, a penicillin V-conjugated PEG-PAMAM star polymer was developed in which the penicillin V was modified with PEG and then conjugated to PAMAM (polyamindoamine) dendrimer 117. This ester-containing penicillin-conjugated dendrimer was incubated within *S. aureus* cultures, where the ester bonds hydrolysed and released penicillin exhibiting antimicrobial effects. Another example, Nguyen and colleagues reported a novel polymeric nanoparticle based on gentamicin-NONOate (nitric oxide donor) co-conjugated polymer, which is capable of releasing both gentamicin and nitrous oxide gas, with a synergistic effect on the elimination of biofilms and planktonic bacteria **(Figure 5)** 118.

### Polymeric nanoparticles-based theranostics against bacterial infection

**Figure 6:**
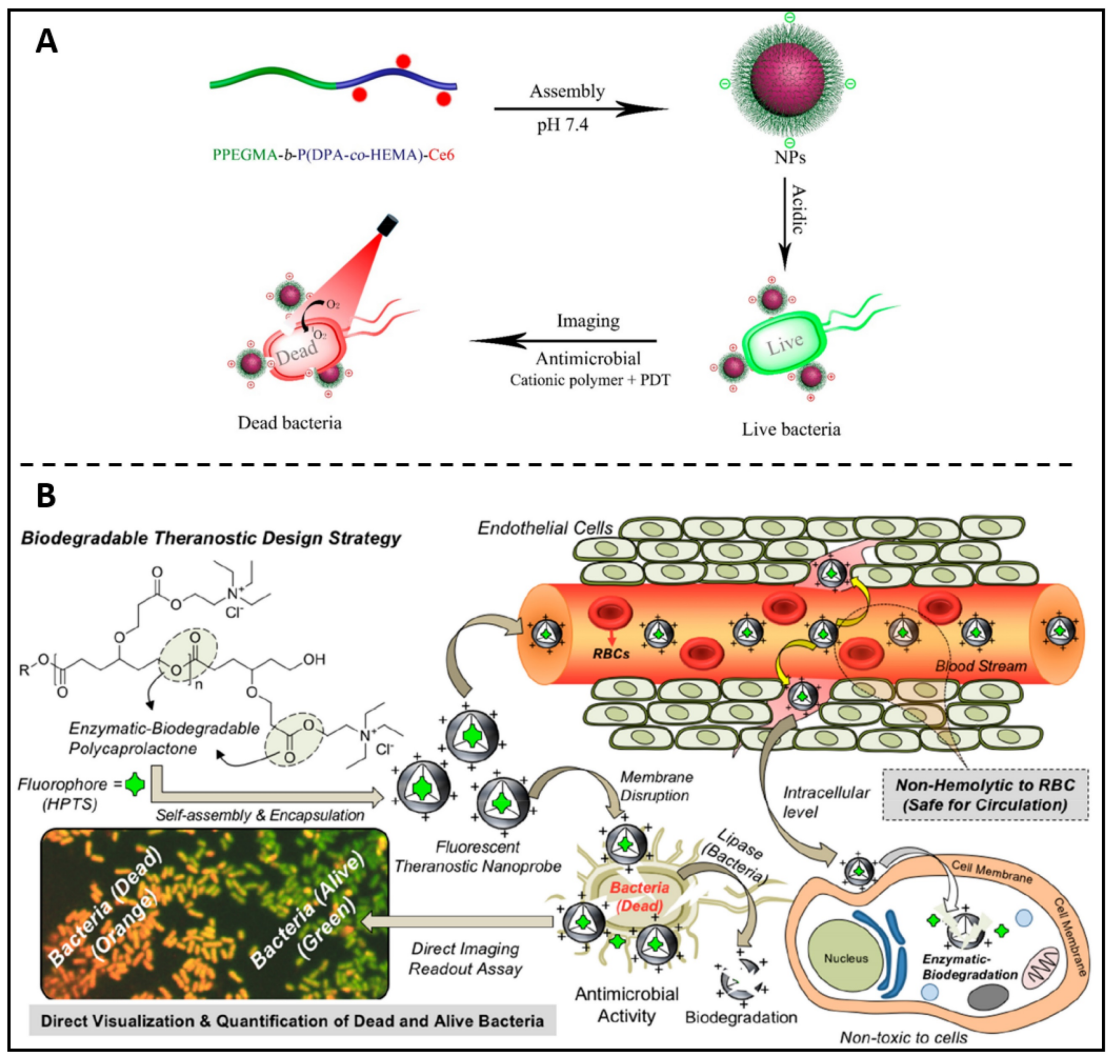
** (A)** Schematic illustration of the PPEGMA-b-P(DPA-co-HEMA)-Ce6 nanoparticles as theranostic antimicrobial agents. Copyright © 2018 The Royal Society of Chemistry** (B)** Theranostic fluorescent cationic polymeric nanoprobes for assessing antibacterial activity are shown in a diagram. Copyright © 2020 American Chemical Society

Following the concept of antibiotic-polymer conjugates, Liu *et al.* replaced the antibiotic portion with the photosensitiser Ce6 and in this way prepared self-assembled polymer nanoparticles with therapeutic properties 119. The self-assembled PPEGMA-b-P(DPA-co-HEMA)-Ce6 nanoparticles were able to bind bacteria efficiently via cations and enable fluorescent bacterial imaging at acidic pH **(Figure 6A)**. In addition, PPEGMA-b-P(DPA-co-HEMA)-Ce6 NPs can achieve good antibacterial effects by photodynamic therapy at acidic pH conditions. Ghosh and colleagues developed a biodegradable fluorescent theranostic nanoprobe based on anionic biomarker HPTS-loaded cationic-charged PCL nanoparticles, for calculating antimicrobial activity quantitatively while simultaneously visualizing **(Figure 6B)** 120. This theranostic nanoprobe solely functions as an indicator to significantly increase living *E. coli*'s (*Escherichia coli*) green-fluorescent emissions, with no antibacterial activities. After activation by a lipase enzyme or lysosomal esterase enzyme, the theranostic probe disrupts membranes of *E. coli*, allowing the co-staining of the nucleus with propidium iodide (red fluorescent). Consequently, green and orange markers, respectively, might be used to discriminate between alive and dying bacteria. These positive findings have inspired polymer chemists to develop new biocompatible polymers with advanced features, such as self-assembled cationic nanoparticles with copolymers which could destabilize in acidic biofilm microenvironments and release antimicrobial farnesol to disrupt * S. mutans (Streptococcus mutans)* biofilms 121, and conducting polymeric nanoparticles loaded with chloramphenicol which act as an intelligent nanotheranostic electro-responsive platform to real-time monitoring antibiotic release and suppressing the development of *E. coli* and *S. sanguinis* (*Streptococcus sanguinis*) 122.

**Figure 7:**
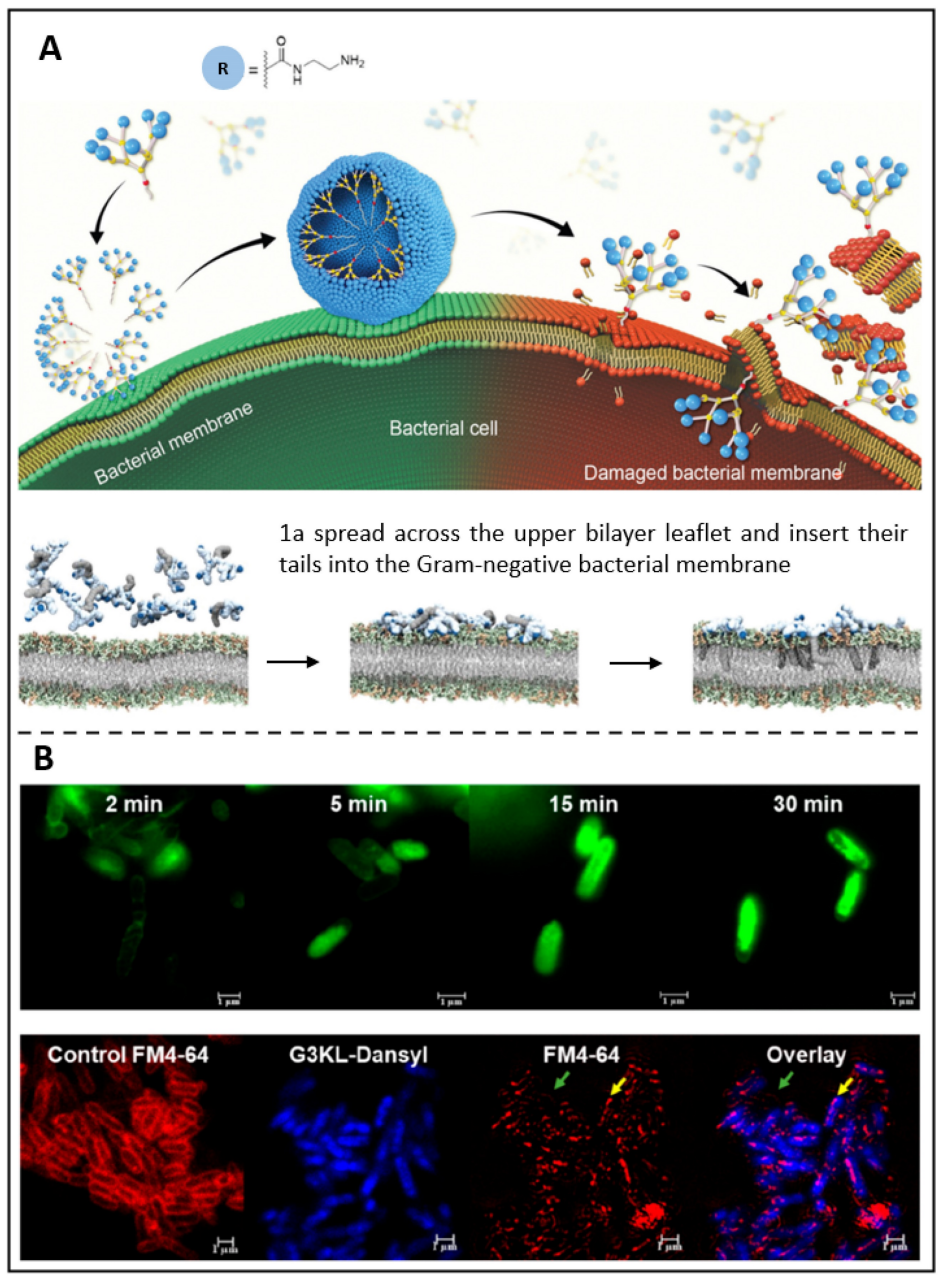
** (A)** A schematic presents the antibacterial activity of amphiphilic dendrimers; Molecular dynamics simulations demonstrate the interaction between dendrimer 1a and bacterial membrane. Copyright © 2022 The Royal Society of Chemistry **(B)** Super-resolution confocal microscopy photos of *P. aeruginosa* exposed to G3KL-Fluo; Confocal microscopy of *P. aeruginosa* treated with G3KL-Dansyl. Copyright © 2019 American Chemical Society.

Dendrimers having various charged terminals, such as primary amine (1a), tertiary amine, guanidine, or carboxylate groups, have recently been synthesized by Dhumal *et al.* **(Figure 7A)** 228. Among these terminals, electrostatic interactions between these terminals cause positively charged 1a dendrimers to move and adhere to oppositely charged membranes, where they enrich and dynamically self-assemble into nanoscale supramolecular components. These nanocomponents then quickly insert hydrophobic tails into the bacterial cytosol and induce cytolytic cleavage, which results in strong antibacterial activity. Such peptide dendrimers have been discovered to drastically reduce the development of Gram-negative bacteria, according to research by Gan *et al.* 123. So, in their recent work, fluorescence-labelled peptide dendrimers containing fluorescein (G3KL-Fluo, **Figure 7B above**) or dansyl (G3KL-Dansyl,** Figure 7B below**) were reported. These two dendrimers bind to the bacterial surface, causing it depolarized and permeabilized while disrupting the external leaflet and inner layer. Moreover, G3KL could bind to LPS (lipopolysaccharide), preventing macrophages from releasing LPS-induced TNF-α. It was shown that G3KL is a potent membrane-disturbing antibacterial peptide that has the potential to be an antimicrobial theranostic nanoplatform.

The physicochemical properties, diameter, and shape of polymeric nanoparticles are among the most critical factors to consider when designing nanomedicine. Polymer nanoparticle characteristics can be significantly influenced by variations in the properties of polymer monomers, especially *in vivo*, leading to differences in performance, including circulation times, biodistribution, metabolic behaviours, and other pharmacodynamic effects. In addition, these polymeric materials' quality standards and economic effects need to be addressed during scale-up production. These considerations are fundamental to generating nanomaterials that can successfully track infections in vivo and remain the most critical issues to be addressed in the field.

## Metallic Nanoparticles

### The antimicrobial mechanisms of metallic nanoparticles

Metallic nanoparticles possess a range of advantages such as broad antibacterial spectrum, high antibacterial activities, good biocompatibilities, durable stabilities and non-resistance. Silver 124-127, gold 127-129, copper oxide 130-132, zinc oxide 133-135, iron oxide 136-138, titanium dioxide 139-141, and magnesium oxide nanoparticles 142-144 have all been frequently documented as antibacterial substances. The antibacterial mechanism of these nanoparticles is mainly: 1. electrostatic adsorption and influences the bacterial membrane and cell wall function; 2. the destruction of bacterial enzymes, proteins, and DNA structure.

Most metallic ions, whether released from pure metallic nanoparticles or metal oxide nanoparticles, can tightly accumulate onto the bacterial surfaces due to their opposite charges, influencing the permeability of the membrane, wrecking the bacterial electron and resource transport systems; on the other hand, causing the destruction of the inherent components of the membrane, resulting in bacterial membrane rupture, cytoplasmic outflow, and ultimately the bacteria death. When the metallic ions penetrate the bacterial membrane and enter the bacterial cell, they combine with the sulfhydryl groups on the protein surface to coagulate the proteins and interfere with the action of enzymes and proteins, thereby depriving the cell of its ability to divide and proliferate. For example, the Ag^+^ and Zn^2+^ reacts with -SH to form a stable S-Ag and S-Zn bond that inactivates enzymes associated with oxygen metabolism in bacteria, which results in the disruption of ATP (adenosine triphosphate) synthesis.

In addition, metallic ions, such as Ag^+^, Zn^2+^, Cu^2+^, which can encourage the formation large amounts of ROS in microbial cells, leading to oxidative stress. Additionally, metal oxide like ZnO NPs and TiO_2_ NPs can activate electron-hole pairs to produce ROS when irradiated by light or UV light. These ROS bind to DNA and replace the hydrogen in the double helix structure of DNA molecules, thus leading to the deformation of the structure of bacterial DNA molecules, inhibiting the synthesis of DNA, RNA, and protein, and rendering the bacteria inactive.

### Metallic nanoparticle-based antimicrobial strategies

There are some metallic nanoparticle-based strategies, mainly based on silver, that have been successfully marketed. AgTive® comprises central venous catheters impregnated with AgNPs to enhance bactericidal properties 145. When in contact with bodily fluids and intravenous solutions, AgTive® releases silver ions to reduce bloodstream-borne infections. Similarly, Acticoat™ is a nanocrystalline silver wound covering that offers a powerful antimicrobial barrier to a broad spectrum of wound microorganisms 146-148. This nano-formulation has been demonstrated to inhibit biofilm development in *A. baumannii* (*Acinetobacter baumannii*) and* P. aeruginosa* models by more than 90%. Salomoni and colleagues evaluated the antimicrobial efficacy of commercial 10 nm AgNP on multi-resistant *P. aeruginosa* strains found in hospitals 149. The findings revealed that all strains were vulnerable to a 5 g/mL AgNPs solution, revealing the potential clinical application for nano-silver against pathogenic bacteria. These silver nanoparticles' biological effects and possible toxicity, however, might vary depending on their size and structure, as with other nanoparticles 150-153. For example, elongated wire AgNPs elicited adverse effects on A549 cells, whereas similar concentrations of spherical particles did not produce similar adverse effects 154. Furthermore, it has been found that AgNPs exhibit cellular cytotoxicity through the generation of ROS in eukaryotic cells, triggering caspase-1 activation that regulates pyroptosis and other forms of cell death 155, 156.

An alternative metal that has been widely examined in nanoparticulate form is gold. In addition to the enhanced safety and toxicity profile of gold over silver, it offers many adaptable features. At a mechanistic level, gold can alter the membrane potential of bacterial cells, thus abrogating adenosine triphosphate synthase activity, resulting in reduced energy production in the cell 157. It has also been demonstrated that AuNPs can impede tRNA binding towards the ribosome, resulting in protein synthesis blockade 158, 159. Furthermore, it is thought that the large surface area elicited by small AuNPs can enhance the surface reactivity of the particles, resulting in the promotion of charge-dependent interaction between AuNPs and bacteria, disrupting the cell wall 160. The potential of these broad non-targeted mechanisms of action was emphasized by Li *et al.;* cationic AuNPs were shown to be capable of inhibiting the development of a variety of bacteria, including MDR (multidrug resistance) strains and MRSA, without inducing bacterial resistance over 20 generations.

**Figure 8:**
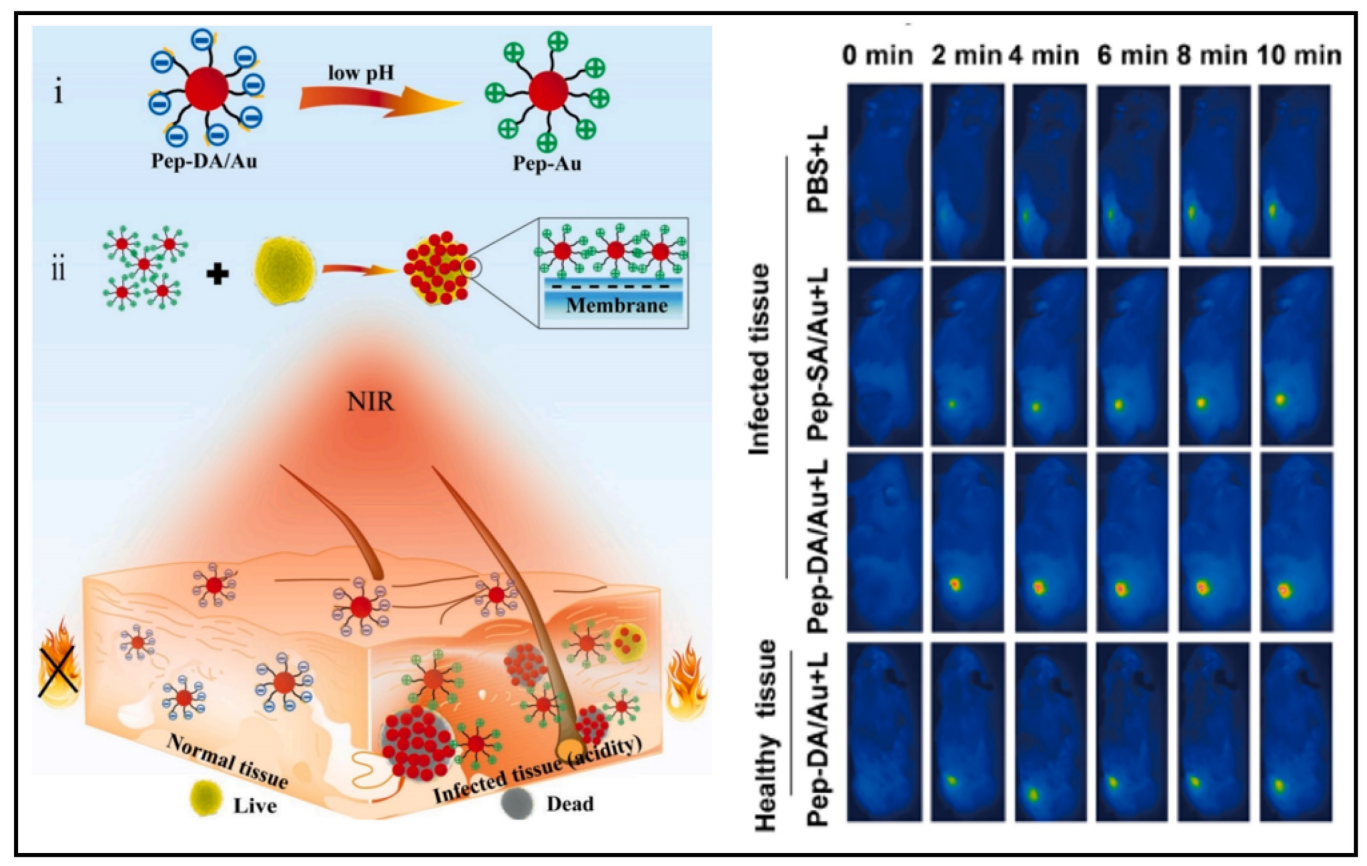
A schematic presents AuNPs for bacterial-selective therapy. Copyright © 2020 Elsevier B.V.

AuNPs have also been used to generate antimicrobial effects through photothermal therapy. Hu and colleagues developed a gold nanoparticle exhibiting a self-adaptive surface to target biofilms, which could inactivate the biofilm-resident MRSA upon near-infrared light irradiation 161. Wang *et al.* synthesized an acidity-triggered gold nanoparticle with the charge-convertible ability (named as Pep-DA/Au)** (Figure 8)** 162. In a typical physiological environment, the nanoparticles were negatively charged; however, when they reached the bacterial infection zone, the acidic environment caused DA (dimethylmaleic anhydride) to hydrolyze, exposing the amino group of Pep, causing the negatively charged NPs to become positively charged and thus accumulated onto the bacterial surface. SEM images showed that the clustered AuNPs generated heat, specifically inactivated bacteria when exposed to near-infrared light. From the infrared thermography, the abscess area injected with Pep-DA/Au exhibited a rapid temperature elevation up to > 50 ℃ under irradiation with the 808 nm laser, which was sufficient to inactivate the bacteria **(Figure 8 Right)**. It suggested that Pep-DA/Au caused hyperthermia only in areas contaminated with bacteria and did no harm to normal organs.

Another interesting way to exploit AuNPs in antimicrobial applications is by conjugating antibiotics onto their surface. Because of the enormous surface area, physiologically relevant drug concentrations could be functionalized onto the particles, resulting in drug carrier/drug delivery systems. For example, Saha *et al.* functionalized AuNPs with ampicillin, streptomycin, and kanamycin 163. These antibiotic-conjugated nanoparticles exhibited greater bactericidal activity towards three pathogenic strains, *E. coli*, *S. aureus,* and *M. luteus* (*Micrococcus luteus*), implying that AuNPs conjugated antibiotics were more potent than free drugs.

The utilization of metallic nanoparticles with broad mechanisms of action has exciting potential for the treatment of both antibiotic-resistant pathogens and preventing further development of drug resistance in the future. However, toxicity remains a significant concern, with metal particles (and by-products) linked to nephrotoxicity and immunotoxicity. Furthermore, substantial progress is needed in understanding the uptake, distribution, metabolism, excretion, and toxicology of such metallic formulations generated under appropriate production circumstances.

### Metallic nanoparticle-based bacterial diagnosis strategies

Metallic nanoparticles have been investigated in the context of pathogen tagging and tracking, in addition to their potential as antibacterial treatments. Properties including a sizable particular surface area and the opportunity for external modification highlight Ag/AuNPs as potential candidates for enhancing signaling intensity, accelerating signal transduction, and providing an accurate signal readout for detecting and visualizing microorganisms 164. An example of this is the development of mannose-Au nanodots, which have been shown in preclinical models to selectively and quantitatively label infective *E. coli* by virtue of mannose receptors on the bacterial membrane surface 165. Currently, only a commercialized AuNP for diagnosis is available within the infectious diseases domain, Verigene® 166. In approximately three hours, this *in vitro* test can identify the genus and species of Gram-positive microorganisms in blood, including the resistant bacterial strains. This Gram-negative blood culture nucleic acid test uses an AuNP-based hybridization mechanism to achieve amplification. Briefly, the bacterial nucleic acids are extracted from blood culture media and hybridized to specific oligonucleotides on a microarray slide. Then functionalized AuNPs further hybridize to the poly-A tail of the captured nucleic acids, followed by amplification. Finally, the scattered light of silver-amplified AuNPs is imaged and analysed by Verigene Reader.

Metallic nanoparticles, especially MNPs (magnetic nanoparticles) with exceptional superparamagnetic properties, have the ability to be modulated by an outside magnetic field and developed as MRI agents in the field of clinical diagnostics 167, 168. MRI is a diagnostic technique that is well established for in vivo use and clinical adoption, delivering a high spatial resolution while avoiding hazardous radiation related to contemporary therapeutic modalities. Table 1 lists the MNP formulations that the FDA has approved as MRI contrast agents so far for clinical uses.

MNPs have now been evaluated for diagnostic purposes in a variety of inflammatory disorders. For example, in Baraki and colleagues' work, neutrophil granulocytes were firstly labelled with FeraTrack^TM^ superparamagnetic iron oxide NPs (passively taken up through the inherent phagocytic phenotype of these cells) and then injected into *S. aureus* infected rats. MRI clearly demonstrated granulocyte migration to infected tissue 169. Such an approach cannot only facilitate diagnosis of the infection site, but also offer a physiological perspective into the infection process. Prior to *in vivo* inoculation, Hoerr *et al.* used a similar technique to tag *S. aureus* with iron oxide nanoparticles, thereby enabling real-time surveillance of the infected tissues and, subsequently, the host inflammatory response 170.

**Figure 9:**
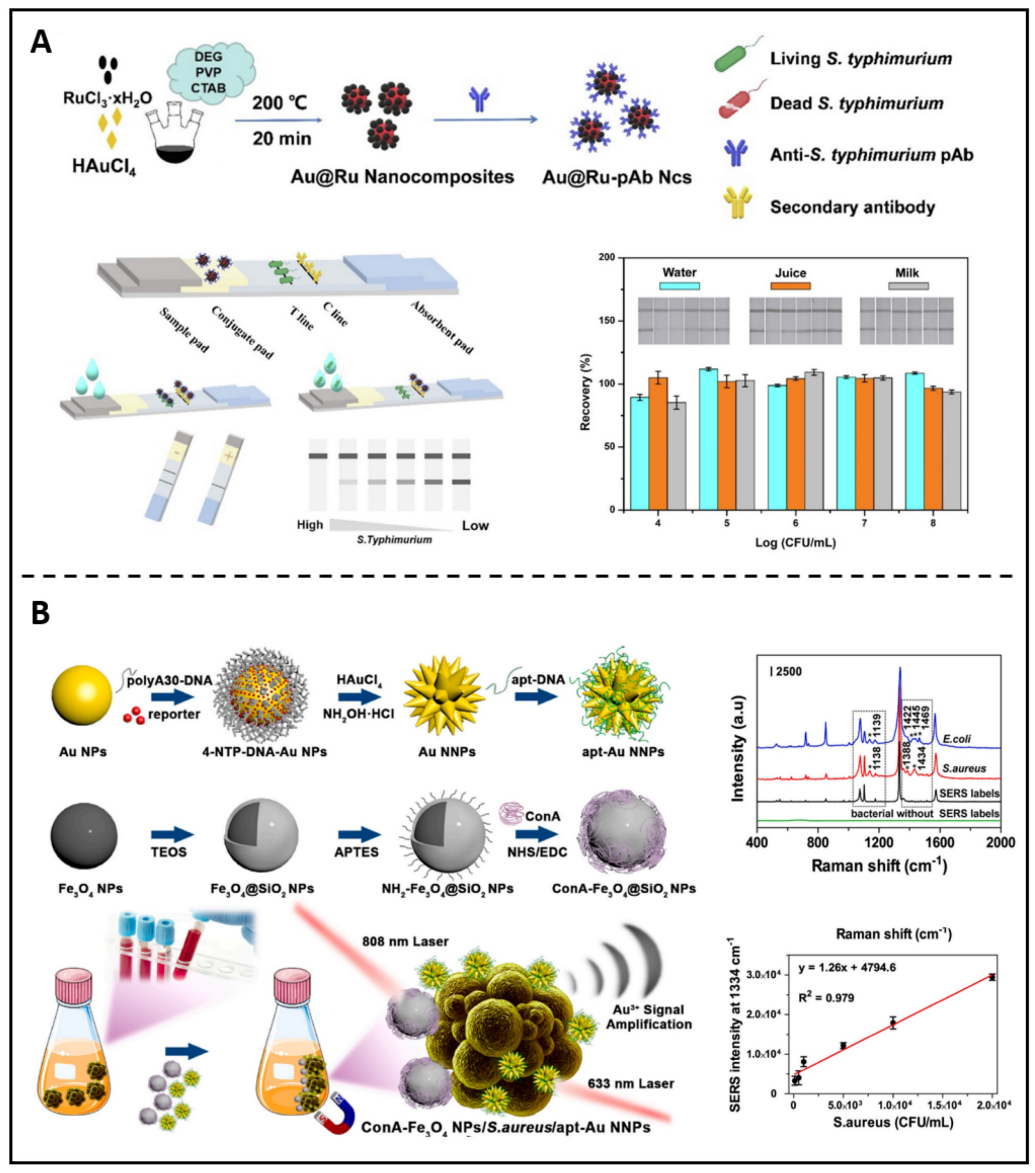
**(A)** A diagram showing the manufactory process of Au@Ru-pAb nanocomposites; The detection of *S. typhimurium* in different samples. Copyright © 2022 Elsevier Inc. **(B)** A schematic presents the synthetic procedures of apt-Au NNPs and the SERS spectra of Au NNPs labels for distinguishing different bacteria strains. Copyright © 2022 Elsevier B.V.

Other metallic nanoparticle-based diagnostic approaches are still in preclinical development. For example, the LFIA (lateral flow immunochromatographic assay) is a typical sensitive diagnostic technique based on antigen and antibody immune reactions because of its superior visibility, reliability, and operability. It has become a standard method in many fields, including medical assessment, environmental impact assessment, and food security requirements. Ji *et al.* recently described an ultra-sensitive probe based on Au@Ru Ncs linked with an anti-*S. typhimurium* antibody and constructed as a retinal marker for the LFIA **(Figure 9A)** 171. The practicability and particularity of the presented biosensor were verified by recognizing various samples. Later investigations proved that the immunoassay method based on Au@Ru could precisely identify *S. typhimurium* in the target samples. SERS (Surface-enhanced Raman scattering), a potential approach based on the LSPR (localized surface plasmon resonance) of plasmonic NPs, has emerged as an attractive non-destructive option for bacterium identification. Huang *et al.* created a unique approach based on sandwich nanocomposites that can be used for pathogenic strains' differentiations and quantifications using SERS and ICP-MS (inductively coupled plasma mass spectrometry) **(Figure 9B)** 172.

### Metallic nanoparticle-based anti-bacterial theranostics

**Figure 10:**
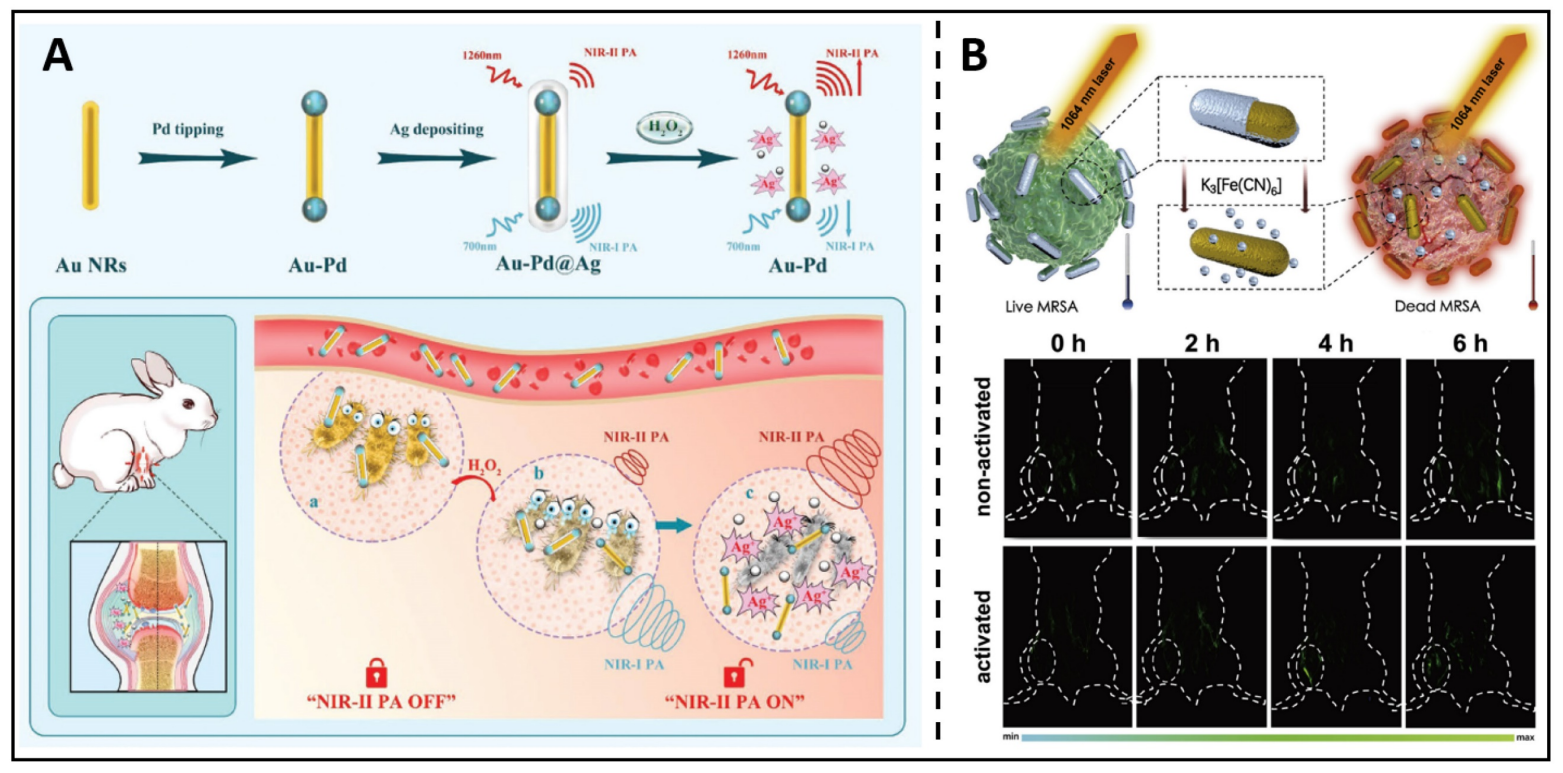
** (A)** A schematic presents the Au-Pd@Ag nanoprobe applied in bacterial infection. Copyright © 2020 WILEY-VCH Verlag GmbH & Co. KGaA, Weinheim. **(B)** Schematic diagram and the NIR-II triggered PA images for *in situ* Ag^+^ release tracking. Copyright © 2020 Elsevier Ltd.

From another indirect perspective, excessive ROS generation, such as H_2_O_2_, is the pathological underpinning of inflammation caused by bacterial infection. Therefore,* in situ* H_2_O_2_ levels are a reliable indicator, and the real-time monitoring of H_2_O_2_ levels can be used to evaluate the progression of the disease and improve therapeutic outcomes. Therefore, theranostic NRs were developed by Ye *et al.*, which utilised Pd-tipped gold nanorods incorporated in an Ag shell 173. This outer silver shell shifts the maximum absorption peak of these Au-palladium nanorods. It extends to 1260 nm, allowing PA imaging at 700/1260 nm to correctly measure H_2_O_2_ in a bacterial infection mouse model **(Figure 10A)**. Moreover, the formation of Ag ions from the selective etching of the nanorod shell by H_2_O_2_ at the infection site served as a broad-spectrum antibacterial agent, eradicating microorganisms with no resistance. Non-invasive diagnostic strategies, such as PA (photoacoustic) imaging, are always combined with the antimicrobial effects of metal NPs, such as antimicrobial ion release and phototherapeutic effects, acting as an integrated diagnosis and treatment. Mei *et al*. developed a therapeutic system using an activable near-infrared light source, which uses Au/Ag nanorods combined with photochemistry to realize in situ dynamic observation of MRSA infection 174. Au/Ag NRs can be triggered rapidly, and the nanorods can constantly separate silver ions for a while after triggering, thus eliminating MRSA and gradually increasing the photothermal and PA capacity of the NIR II region. After cooperative treatment, the signal difference between tissues can be up to 20 times, which helps monitor the silver ions' release process in real-time **(Figure 10B)**. Under 1064 nm laser irradiation, free Ag^+^ and near infrared-II PTT (photothermal therapy) can exert the effect of "1+1>2". After activating Au/Ag, the temperature rose dramatically from 36.0 ℃ to 51.0 ℃, successfully eliminating the MRSA; inactivated and other control groups, on the other hand, showed a limited temperature considerably underneath the thermal criterion for killing MRSA. Such alloyed nanoparticles showed great potential for an antimicrobial nanotheranostic platform.

While metallic nanoparticles are desirable candidates for theranostics due to their various broad-spectrum antimicrobial mechanisms and their own imaging properties, as well as the large-scale production capacity of metallic nanomaterials, which can be standardized in terms of quality, the *in vivo* distribution and metabolism of metallic nanoparticles and metallic materials has remained unclear, which leads to further difficulties in clinical trials and commercialization.

## Quantum Dots

### Quantum dots as antimicrobial therapeutics delivery systems

With controllable properties such as surface modification, extensive surface area, high molecular loading ability, and capability to cross physiological barriers, QDs (quantum dots), a diverse chemical semiconductor, have become promising materials in DDS for intracellular delivery of micro molecular compounds, peptides, and nucleic acids. For example, a Zn-Rif (zinc-rifampicin) complex was synthesized and loaded into transferrin-conjugated QDs to achieve an efficient DDS for improved anti-tuberculosis therapy 175. Compared with free RIF and Zn-RIF, these drug-loaded QDs exhibited higher anti-mycobacterial activity, where it was discovered that infected dendritic cells absorbed the QDs effectively, effectively to the site of infection. Given the absence of heavy metals in the structure, ZnS QDs have a long emission lifespan, improved water solubility, and low toxicity, which allows them to be widely used in biological systems. A pH- triggered QDs drug delivery system, described by Mandani *et al.*, was proposed for the antimicrobial application of gentamicin 176. In this study, MSNs (mesoporous silica nanoparticles) were chosen as gentamicin carriers, and MPA-ZnS QDs were used to encapsulate the nanopores and prevent the premature release of the drug. Under acidic microbiological surroundings, MPA-ZnS QDs dissociated, MSN pores opened, and gentamicin dispersed to the outside.

Aside from the capacity to obtain high drug loading, researches have indicated that the customizable nature of semiconductor nanomaterial electrical characteristics provides an avenue for eliciting particular redox disturbances. Courtney *et al.* showed that small QDs, tuned through size-dependent quantum confinement (2-4 nm), generated specific LARS (light-activated redox species), to trigger pathogen cell death and eliminate various clinical MDR isolates 177. In their theory, CdTe-2.4 has a lower band gap than other metallic oxide materials but has superior oxidation and reduction potentials to produce redox-active species in the presence of light. Reflected in the experiments, it was shown that in the system of co-incubation of MDR clinical isolates with CdTe QDs, a robust growth inhibition effect was obtained for each clinical MDR strain when there was light. In subsequent validation experiments, they found that CdTe-2.4 (non-excited state) showed no phototoxic effect in co-cultures of *E. coli* and HEK293T; instead, under light, CdTe-2.4 suppressed *E. coli* growth without causing damage to HEK293T cells, giving evidence of concept regarding the application of bacteria-specific phototoxic nanomaterials in therapeutic strategies.

### Quantum dots as bacterial labelling tools and their potential as theranostic platforms

For the purpose of diagnosing bacterial infections, ensuring an early diagnosis, and monitoring the development of the illness over time, *in vivo* bacteria surveillance is crucial. A NIR-II surveillance technique based on fluorescent PbS QDs for real-time diagnosis of bacterial infection *in vitro* and *in vivo* was developed by Feng *et al*. 178. The imaging potential of PbS QDs was investigated in a bacteria-infected nude mouse. The fluorescence signal recorded at the location of bacterial infection was typically more significant following the injection of PbS QDs than the control group. PbS QDs were found to quickly tag bacteria at the point of bacterial infection, displaying an instantaneous highly fluorescent signal.

Both Gram-positive and -negative bacterial surfaces contain charged moieties (e.g., teichoic acids and LPS, respectively) which result in a net negative charge on the bacteria's surface. Therefore, the application of cationic QDs has been examined for their ability to label pathogens 179. For example, Arshad *et al.* developed CdSe/ZnS QDs to identify pathogenic *V. harveyi* (*Vibrio harveyi*) using fluorescence microscopy. The electrostatic interaction between QDs and microbial surface successfully contributed to the recognition of bacteria and bacteria-host fibroblast L929 cells 180. In addition to this “passive” electrostatic interaction, an “active” binding approach using high-specificity peptide targeting the bacteria has also been explored. In this method, SiO_2_ stabilized ZnO QDs were conjugated with a potent *S. aureus* targeting peptide UBI29-41 and further modified with a NIR dye MPA to detect pathogenic infection. This nanoparticle was subsequently functionalized with methicillin, transforming it into a theranostic nanoparticle for identifying and treating *S. aureus* infection.

**Figure 11:**
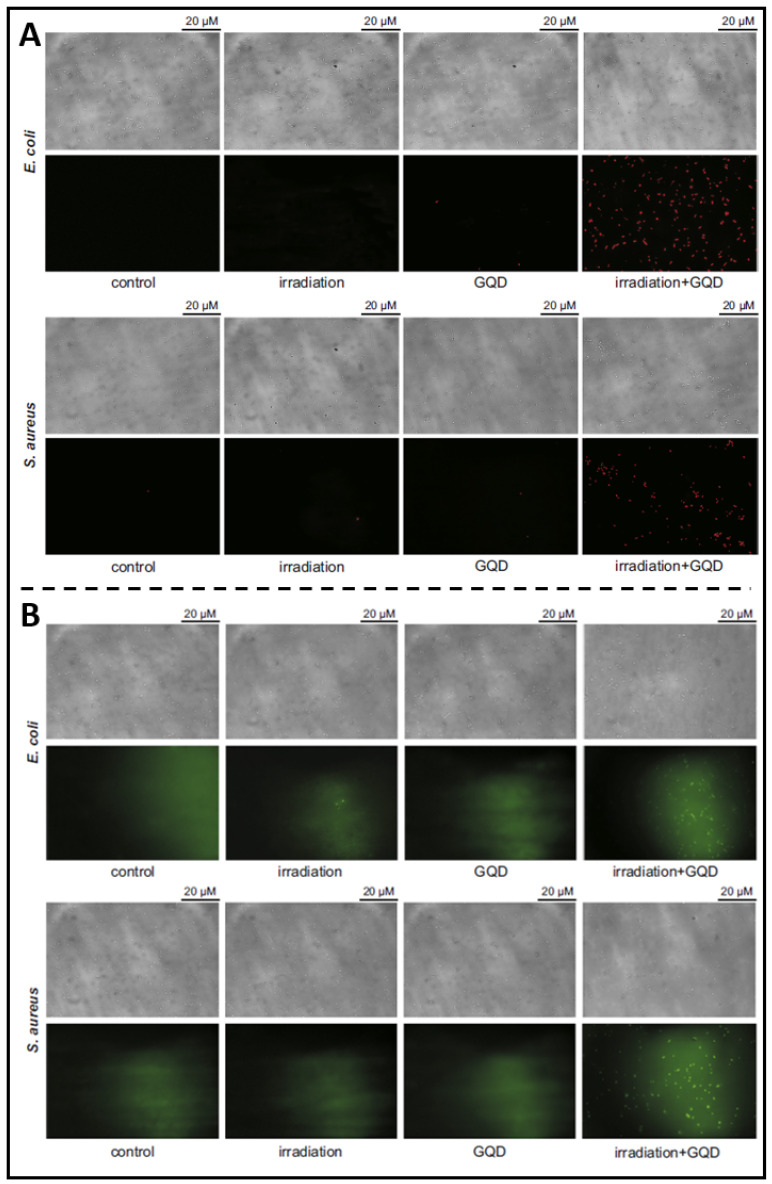
** (A)** Fluorescence microscopy showing the viabilities of *E. coli* and *S. aureus* treated with G-QDs; **(B)** Fluorescence microscopy showing that photoexcited G-QDs induce oxidative stress in bacterial cells. Copyright © 2014 Elsevier Ltd.

The development of carbon nanomaterials has led to a surge in interest in C-QDs (carbon quantum dots). Among carbon materials, graphene is a honeycomb-like single layer of carbon atoms. Therefore, G-QDs (graphene-based quantum dots) possess a vast surface area and outstanding thermal stability with uncomplicated production, high fluorescence activity, resistance to photobleaching, good solubility, and biocompatibility. Due to these favourable characteristics, G-QD is more suitable as a candidate material for non-toxic bioimaging or biosensing agents. For example, as reported in Ristic *et al.* previous article, suspended G-QDs in liquid could be excited by blue light (465-475 nm) to produce ROS through energy and electron transfer to molecular oxygen, thereby killing cancer cells 181. They further prepared G-QDs and examined their photodynamic antibacterial effects under the irradiation of blue light on MRSA and *E. coli*, demonstrating photodynamical disruption on bacterial cell membrane integrity **(Figure 11A)** and indicating that G-QDs entered the damaged bacteria to produce additional ROS amounts **(Figure 11B)** 182. Moreover, Sattarahmady *et al*. assessed the photothermal antibacterial action of C-QDs on the survival of methicillin-resistant and wild *S. aureus in vitro* 183. PTT based on C-QDs demonstrated a targeted thermal ablation approach that induces irreversible bacterial death. These findings demonstrate the advantages of carbon-based QDs for pathogen identification and treatment, highlighting their use as candidate nanoparticulate theranostics.

In recent years, studies have shown that quantum dot materials have excellent properties as antimicrobial materials, but it is worth noting the toxicity of QDs made from inorganic materials, semiconductor materials, and carbon materials in living organisms and the potential for this toxicity being further amplified by quantum dotting, thus limiting the biological applications of these materials.

## Carbon Nanotubes

### Carbon nanotubes as delivery platforms and its broad-spectrum antibacterial property

When it comes to carbon nanomaterials, CNTs (carbon nanotubes) are being researched as possible antibacterial DDS platforms. CNTs with positively charged ends on their surface have been found to bind to G- bacteria, such as *E. coli*, preferentially 184. Based on this, Jose Rojas-Chapana *et al.* developed water-dispersible multi-walled CNTs which could form temporary nano-channels across the cell wall, facilitating plasmid delivery into *E. coli*, paving the way for further development of targeted and controlled delivery of therapeutics towards pathogenic bacteria 184. When CNTs come into contact with bacterium cells, they cause cellular membrane disruption, which results in high antibacterial action. For example, the fullerenes nanoparticles induce cell membrane breakdown and/or DNA cleavage due to the particle's hydrophobicity, which can easily interface with cellular membrane lipids 185. Moreover, because of their robust ROS generation, CNTs display broad-spectrum efficacy against pathogenic organisms upon illuminating, despite medication resistance status.

### Carbon nanotubes as potential deep-tissue bacteria-specific imaging materials

Due to their good Stokes' shift and flexibility of targeting agents, CNTs can be used as superior targeting materials to realize fluorescence imaging of organisms, lesions, and vascular pathways 186, 187. Compared with the traditional dye and the first-window near-infrared imaging, as the potential fluorescence carrier of the second-window near-infrared imaging, SWCNTs (single-walled carbon nanotubes) can achieve better penetration depth in biological tissues 188. To differentiate between F'-negative (DH5α) and F'-positive (JM109) E. coli strains, Bardhan *et al.* created a functional SWCNTs complex (M13-SWNTs) utilizing transgenic M13 bacteriophage 189. After further modification, M13-SWCNT successfully combined with antimicrobial antibodies to achieve the active targeting of bacterial infection. In the detection of *S. aureus* muscle infection and endocarditis infection, the fluorescence signal increased by several orders of magnitude, which effectively proved its possibility in deep tissue detection.

### Carbon nanotubes as potential antimicrobial theranostic materials

Since the SWNTs have therapeutic and imaging properties, the antimicrobial theranostic potential was examined by Kotagiri and colleagues 190. They synthesized stealth DS (dextran sulfate) coated SWCNTs that functioned with an antibody targeting *S. aureus.* The DS-SWCNTs were shown to selectively target the pathogenic bacteria, as well as non-invasively purge the pathogens after NIR laser irradiation.

The three-dimensional structure formed by the unique alignment of carbon atoms provides a strong guarantee of drug delivery, while the photothermal and imaging properties generated by the special alignment make carbon nanotubes a powerful potential for theranostics materials.

## Metal Organic Frameworks and Covalent Organic Frameworks

Metal organic frameworks (MOFs) and covalent organic frameworks (COFs) are innovative medically relevant nanomaterials that have lately piqued the curiosity of researchers worldwide. MOFs and COFs have been proposed for a variety of applications, including isolation, catalysis, sensors, and drug carriers.

### MOFs and COFs as potential antimicrobial theranostics based on deliver properties

They are appropriate for designing as nanocarriers because of their qualities, such as vast surface areas, biodegradable and biocompatible architectures, and remarkable possibilities, such as cell-specific targeted delivery and transportation across obstacles. In addition, due to their high porosity, MOFs and COFs exhibit remarkably high loading capacity when used as carriers for antimicrobial drug delivery platforms (Table 2).

**Figure 12:**
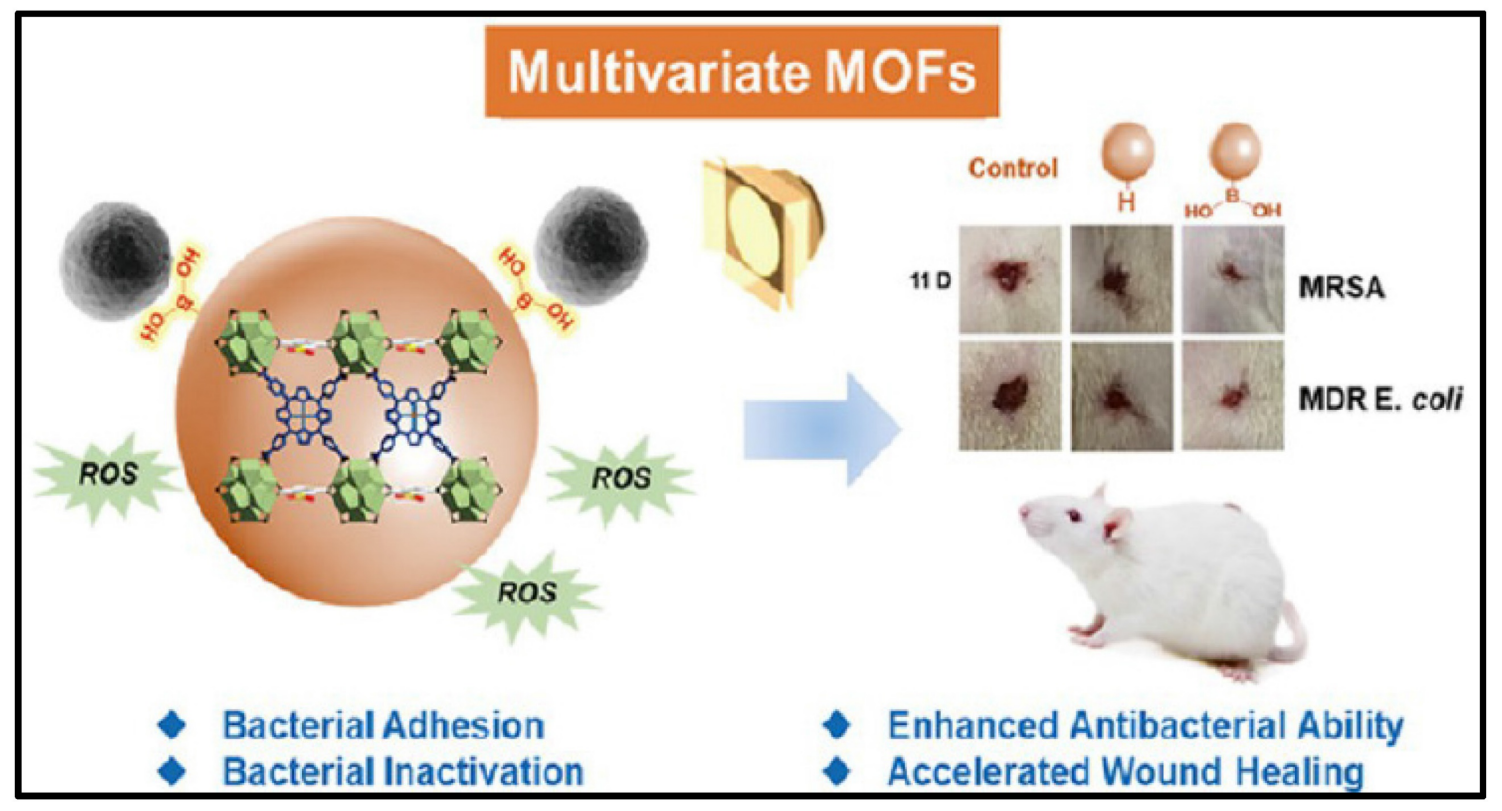
A schematic presents the MOFs with bacterial-binding abilities. Copyright © 2022 American Chemical Society.

Porphyrins and metalloporphyrin dyes can be activated by visible light in the presence of O_2_ to generate O_2_^-^ via photocatalysis. When such photosensitizers are solubilized with the substrate to be oxidized, the reactive oxygen produced is found for many biological applications. MOFs are insoluble in solvents, and when they are stabilized as suspensions, they can adsorb large amounts of molecular oxygen and efficiently produce singlet oxygen in a heterogeneous process. MOFs appear to be suitable candidates for such applications as a result of their extensive surface region and high gas adsorption capability. For example, Chen *et al.* induced photosensitized porphyrin Cu(II) into Zr-MOF and surface modified with bacterial-binding boronic acid ligand **(Figure 12)** 208. TCPPCu-BBDC has been shown to successfully suppress the growth of four different bacterial strains. The colony counts of G+/- bacteria, and their MDR strains, were significantly reduced after 30 minutes of light exposure. In a rat dorsal skin injury model, during the same time span, the TCPPCu-BBDC-treated group with light irradiation outperformed the control group in wound healing. As efficient photosensitizers, COF variants with reticular structures that rely on covalent bonding instead of ligand interactions have recently been created. There are also examples of COFs with excellent photodynamic properties for the inactivation of microbes. Hynek *et al.* reported porphyrin-based COFs with three-dimensional and diamond-like structures 209. The photophysical and photochemical characteristics of the three-dimensional COFs revealed that they were effective in producing O_2_ with visible light irradiation and had robust antimicrobial properties against *P. aeruginosa* and *E. faecalis* (*Enterococcus faecalis*) biofilms. Zhang *et al.* investigated the utilization of a boron-based COF for antibacterial activity 210. The results demonstrated that COF-1, the first boron oxygen compound, could generate considerable quantities of ROS when exposed to visible light and had strong antimicrobial activities against *E. coli* and *S. aureus*. Based to the plate coating findings, all bacteria were eradicated after 120 minutes of co-incubation with COF-1 under daylight. Notably, photodynamic MOFs and COFs are powerful against G+/- bacterial strains via the "delivery" of reactive oxygen species.

### MOFs as potential antimicrobial platforms based on ligand component releasing

It is worth noting that MOFs have been underwent substantial research as reservoirs for antimicrobial agents in the area of controlled drug release. Most MOFs with moderate or weak coordination bonds are unstable in an aqueous environment and several factors in the environment (e.g. pH) can contribute to the collapse of the MOFs structure and release of components. As the basic framework element of the MOF structures, metallic ions and organic monomers are slowly released as the MOFs decompose, exhibiting active antibacterial activity.

Metallic ions, such as Ag^+^, Zn^2+^ and Co^2+^, exert remarkable antibacterial activities by influencing ion channels in the bacterial cell membrane and disrupting proteins and enzymes required for bacterial metabolism. This part has been discussed in the previous section and will not be repeated here. As for the other component, some antimicrobial organic monomers cause fragmentation of bacterial DNA through binding to inorganic salt ions (e.g. calcium and magnesium ions) within the bacteria, as well as through the production of ROS. As the MOFs decompose, these organic antimicrobial monomers are slowly released, thus exhibiting antimicrobial activity. For example, Tamames-Tabar *et al.* reported that a Zn azelate MOF named BioMIL-5 containing the azelaic acid ligand showed synergistic inhibition of *S. epidermidis* after decomposition 211. Restrepo *et al.* reported that a Zn-based MOF containing an antimicrobial 4-hzba (4-hydrazinyl) benzoate ligand demonstrated notable inhibition of the growth and metabolic functions of *S. aureus* 212. According to their report, in the degraded composition of this MOF, the Zn^2+^, which is supposed to have an inhibitory effect, did not exhibit antimicrobial activity due to levels that were lower than the effective inhibitory concentration, but the other ligand, 4-hzba, exerted a remarkable antimicrobial effect.

The photosensitive monomers in the structures of COFs and MOFs, and the porous three-dimensional structures they formed, as well as the antimicrobial metallic ions and organic linkers in their component, make them highly suitable drug currieries with multi-synergistic potentials, being an emerging and promising nanomaterial for theranostics. Although still in their early stages, many teams have created MRI-trackable MOF delivery systems, MRI-trackable photothermal therapy-based drug delivery systems, and core-shell theranostic MOFs. In a nutshell, COFs and MOFs have enormous biomedical promise. These nanocarriers have the potential to usher in an age of targeted drug delivery, precise detection, and theranostic uses.

## Nanostructured Antibiotic/Dye Self-assembly

**Figure 13:**
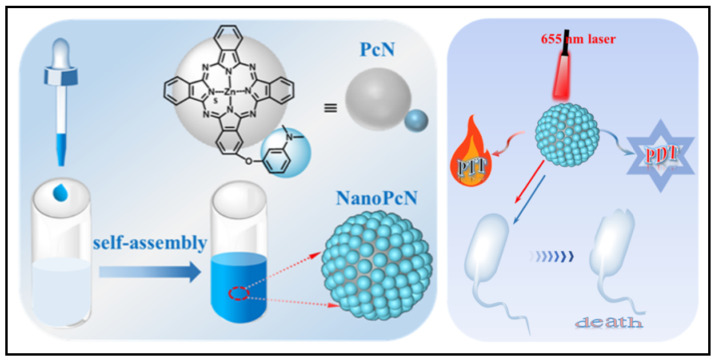
Fabrication and characterization of NanoPcN; Schematic illustration of the antibacterial mechanism of NanoPcN. Copyright © 2022 American Chemical Society.

To combat issues with bacterial infections, researchers have been working to find new antimicrobial compounds, synthesize new materials, and search new approaches. One of the hottest areas of chemistry during the decade is supramolecular chemistry, which focuses on self-assemble systems produced by non-covalent electrostatic, hydrophobic bonding, hydrogen bonding, metal-ligand coordination, van der Waals interactions, and π-π stacking interactions 213. These highly organized nano-scaled supramolecular biomaterials have the potential to create novel theranostics for the treatment of bacteria by combining therapeutics and diagnostics. For example, Xie* et al*. presented a new series of pure theranostic nanomedicines based on ciprofloxacin, in which they linked ciprofloxacin molecules to perfluoroaryl and aryl moieties 214. These conjugated propeller-like ciprofloxacin derivatives, once precipitated into aqueous solution, were easily self-assembled into stable nanoaggregates, which converted these derivative compounds into activated AIE (aggregation-induced emission) luminophores, which were successfully exploited for bioimaging of bacteria; simultaneously, these aggregated ciprofloxacin nanostructures demonstrated strengthened antibacterial activities against both susceptible and drug-resistant *E. coli*. Similar strategies have been adapted to photosensitive nanomaterials for aPDT (antimicrobial photodynamic therapy) and aPTT (antimicrobial photothermal therapy). For example, Li* et al.* synthesized a novel compound 2,4,6-tris-(N,N-dimethylaminomethyl)phenoxy substituted zinc(II) phthalocyanine, named PcA 215. In aqueous solution, PcA undergoes self-assembly to form a nanostructured phthalocyanine, called NanoPcA, which can undergo highly productive ROS generation through the Type I photoreaction, thereby exhibiting potent antibacterial activities. Based on the same conception, recently, Wang and colleagues have proposed another nanostructured modified zinc(II) phthalocyanine, called PcN **(Figure 13)** 216. Self-assembled NanoPcN exhibited remarkable bimodal antimicrobial properties by yielding massive ROS and elevating temperatures upon irradiation.

The potential for treating bacterial infections with supramolecular antimicrobial agents, such as those based on cationic chemical compounds, photodynamic, and photothermal compounds, is enormous. These materials can serve as multifunctional platforms for the unique demands of antimicrobial treatment owing to the incorporation of versatile elements into them. However, the preclinical development of nearly all of these supramolecular antibacterial compounds is still in its early phases. The effectiveness of these compounds against clinically latent bacteria remains to be thoroughly evaluated.

## Challenges and Prospects

### Challenges in large-scale manufacturing

Some important reasons for the challenges in clinical translation of nano-formulations are the structural and physicochemical complexity of the nano-formulations, and the need for a set of manufacturing methods and platforms that can produce scalable nano-formulations and that can consistently and reproducibly yield the same qualitative level of nano-formulation in large quantities. Several basic nanomedicine platforms (liposomes) have been successfully produced at industrial scale utilizing techniques that have been developed that do not require complicated manufacturing procedures or the usage of organic solvents. However, challenges arise when nano-formulations become more complex. Examples include surface alterations that include coatings or ligands, various targeting components, or by encapsulating several therapeutic compounds. These require the incorporation of several ingredients into a single nanocarrier, so numerous phases in the manufacturing process are required, which eventually causes issues with cGMP (current good manufacturing practice), increases production costs and makes QA and QC (quality assurance and quality control) assessment more difficult. Characterisation and validation of more complex nano-formulations are likely to be extremely difficult because of the overwhelming amount of characteristics that need to be addressed, including size, morphology, surface charge, drug encapsulation efficiency, surface coating efficiency, and surface ligands density. Moreover, the batch-to-batch stability of nano-formulations during the production process might lead to considerable differences in their physicochemical characteristics, pharmacokinetic parameters, and pharmacodynamic interactions.

### Biological challenges and perspectives

As previously noted, nanoparticles have demonstrated the most significant therapeutic and diagnostic uses in the battle against bacterial infections by overcoming the pharmacokinetic limits of traditional drug treatments or by merging new technologies and modalities. However, nanoparticle-based drug delivery systems all face a series of problems posed by complex biological barriers that limit the bioavailability and therapeutic efficacy of nanodrugs at specific sites, such as the MPS (mononuclear phagocyte system), nonspecific distribution, cellular internalization, and drug efflux pumps 217-219. The MPS, a phagocytic system mostly composed of monocytes and macrophages in the spleen, lymph nodes, and liver, is one of the most critical components of the nanodrug delivery system 220. The MPS eliminates nanoparticles by phagocytosis, which starts with the opsonization of the nanomaterials, which involves the adhesion of serum proteins. Following protein adsorption, nanoparticles are quickly identified by phagocytes and bind to receptors located on the cell surface, before being ingested, transported to the phagosome, merged with lysosomes, and eventually destroyed.

Consequently, in the case of traditional bacterial infections, several tactics have been used in developing nanoparticles to make them less sensitive to being detected by MPS to perpetuate the retention duration at the infection site. The most straightforward and extensively used method is to modify the surface with hydrophilic polymers, like coating nanoparticles with PEG, to prevent the nanoparticles from aggregation and to hinder protein adsorption and removal by MPS for long-term circulation *in vivo*. Another strategy is to use self-identifying markers to conceal the surface of alien nanoparticles. For example, researchers have used sialic acid from certain pathogens to cover nanoparticles with the expectation that these particles will promote inhibition of immune activation, allowing for increased blood circulation and decreased MPS absorption 221. For example, CD47 peptide is a potential “do not eat-me” marker of “self”, which is observed on certain pathogens that they are able to over-express CD47 on some viruses such as smallpox to evade recognition, strategies based on CD47 marker to modification NPs have been widely investigated in cancer research 222-226. Then based on such phenomena, in the case of bacteraemia and sepsis, could we explore more natural markers and use them as surface modification ligands to reduce phagocytosis of the organism and substantially increase the cycle time* in vivo*?

However, as for specific intracellular pathogens, such as *M. tuberculosis*, *S. aureus,* and *K. pneumoniae* (*Klebsiella pneumoniae*), they are supposed to be recognized, opsonized, and eliminated by the MPS, but developed the mechanisms to keep alive in the MPS, which turns out that we can promisingly employ the opsonisation mechanism onto nanoparticles surface modification and use the difference in marker on the surface of infected MPS cells to explore targeted drug delivery strategies for this class of intracellular diseases.

Furthermore, new biotechnologies for eradicating harmful microorganisms are emerging due to the fast emergence of antibiotic resistance and the lengthy research period for an innovative drug. For example, in 2016, Lehar *et al.* developed an AAC (antibody-antibiotic conjugation) against *S. aureus* (THIOMAB™) that rapidly identifies and labels *S. aureus* circulating in the bloodstream 227. The labelled bacterial-AAC complex is internalized by ADCP (antibody-dependent cellular phagocytosis), which releases a potent antibiotic to inactivate the intracellular pathogen through lysosomal protease-mediated cleavage. Following this work, ACNPs (antibody-conjugated nanoparticles) and genetically engineered AMVs (antibody-presented membrane vesicles) might represent a relatively novel class of strategies based on the combination of bacterial-specific antibodies and nano-DDS to enhance antimicrobial delivery and efficacy. On the one hand, the efficient drug-loading capacity of nanoparticles and surface-enriched antibodies may offer a relatively high DAR (drug-to-antibody ratio) to reduce the toxicity induced by the low DAR of conventional AACs and may enhance the ADCP-mediated internalization between nanoparticles and target infected cells due to the antibody enrichment effect; on the other hand, compared to simple AACs, thanks to the long-standing development of nano-DDS development, antibody nano-DDS can achieve a more precisely controlled release through *in vivo* microenvironmental stimulations and* in vitro* stimulations. Furthermore, in combination with some of the novel antimicrobial ingredients mentioned in the previous sections, such as photosensitizers and sonosensitizers, specific aPDTs targeting certain specific strains of bacteria can be achieved. Another example is the CRISPR-Cas (clustered regularly interspaced short palindromic repeats - CRISPR associated) technology, which has received much attention; by targeting specific bacterial DNA sequences, the RNA-guided nuclease Cas9 is able to destroy DNA by chromosomal cleavage, thus altering the resistance genes of drug-resistant strains and thus eliminating the pathogens. However, the lack of an efficient delivery method to turn this excellent treatment technique into an appropriate antibacterial agent significantly limits its efficacy. In combination with antibody nanodrug delivery systems, we hypothesized that this obstacle could be overcome. Cas9 as an antimicrobial agent might be directly targeted to the target pathogenic bacteria using antibody nano-DDS, leading to an extraordinarily sufficient and specific therapeutic strategy to tackle multidrug-resistant microorganisms.

## Conclusion

In this review, we have outlined the most current developments and research into therapeutic, diagnostic, and theranostic nanomedicines for treating bacterial infections. Challenges for the clinical adoption of such technologies remain, but advances in the production of nanomedicines and a better knowledge of their *in vivo* behaviours and biodistribution will likely make this approach to drug delivery a fascinating proposition for the treatment of problematic pathogens in the future.

## Figures and Tables

**Table 1:** List of magnetic nanoparticles commercialized for MRI contrast agent

Compound	Trade Name	Type	Surface Modification	Indication
Ferristene	Abdoscan®	SPIO	Sulfonated styrene-DVB copolymer	Gastrointestinal
Ferumoxsil	Umirem® Lumirem®	SPIO	Siloxane	Gastrointestinal
Ferrixan	Resovist® Cliavist™	SPIO	Dextran	Liver imaging; Cellular labelling
Ferumoxide	Feridex® Endorem®	SPIO	Dextran T10	Liver imaging; Cellular labelling
Ferumoxytol-7228	Feraheme®	USPIO	Carboxymethyl-dextran	Macrophage imaging; Cellular labelling
Feruglose	Clariscan™	USPIO	PEGylated starch	Blood pool agent; Lymph node angiography

SPIO: superparamagnetic iron oxide; USPIO: ultrasmall superparamagnetic iron oxide

**Table 2:** Carriers, Antibiotics, and Loading Capacities of MOF/COF-based composites as conventional drug delivery platforms in Antimicrobial Applications.

Carrier	Antibiotic	Loading capacity	REF.
**MOFs**			
ZIF-8	Ceftazidime	10.9% DLE	191
ZIF-8	Chloramphenicol	56.8% DLE	192
ZIF-8	Tetracycline	31.2 μg/mg	193
ZIF-8	Physcion	88.72% DLE	194
ZIF-8	Ciprofloxacin	21% DLE	195
ZIF-8	Vancomycin	24% DLE	196
Zn-MOF	Amoxicillin	60.15% EE	197
Zinc-based MOF-5	Metronidazole	539.33 mg/g	198
Zn_2_(bdc)_2_(dabco) MOF	Gentamicin	-	199
UiO-66 MOF	Fosfomycin	-	200
UiO-66 MOF	Ciprofloxacin	84% DLE	201
MOF-53(Fe)	Vancomycin	20% DLE	202
chitosan coated MOF-53(Fe)	Vancomycin	9.87% DLE	203
γ-cyclodextrin-MOF	Florfenicol & Enrofloxacin	42.25 mg/g	204
nanoMOFs	Amoxicillin	36% DLE	205
			
**COFs**			
Pyridine-3-boronic acid COF	Trimethoprim	-	206
ENR-FM-COF-TPU	Enrofloxacin	-	207

DLE: Drug Loading Efficiency; EE: Encapsulation Efficiency
